# Geometric characteristics of stromal collagen fibres in breast cancer using differential interference contrast microscopy

**DOI:** 10.1111/jmi.13361

**Published:** 2024-10-03

**Authors:** Suzan F. Ghannam, Catrin Sian Rutland, Cinzia Allegrucci, Melissa L. Mather, Mansour Alsaleem, Thomas D. Bateman‐Price, Rodhan Patke, Graham Ball, Nigel P. Mongan, Emad Rakha

**Affiliations:** ^1^ Academic Unit for Translational Medical Sciences School of Medicine University of Nottingham Nottingham UK; ^2^ Faculty of Medicine Department of Histology and Cell Biology Suez Canal University Ismailia Egypt; ^3^ Nottingham Breast Cancer Research Centre Biodiscovery Institute University of Nottingham Nottingham UK; ^4^ School of Veterinary Medicine and Science University of Nottingham Nottingham UK; ^5^ Optics and Photonics Research Group Faculty of Engineering University of Nottingham Nottingham UK; ^6^ Department of Applied Medical Science Applied College Qassim University Qassim Saudi Arabia; ^7^ Biodiscovery Institute University of Nottingham University Park Nottingham UK; ^8^ Medical Technology Research Centre Anglia Ruskin University Chelmsford UK; ^9^ Department of Pharmacology Weill Cornell Medicine New York New York USA; ^10^ Cellular Pathology Department Nottingham University Hospitals NHS Trust Nottingham UK; ^11^ Pathology Department Hamad Medical Corporation Doha Qatar

**Keywords:** breast cancer, collagen, DCIS, differential interference contrast microscopy, invasive tumour, stroma

## Abstract

Breast cancer (BC) is characterised by a high level of heterogeneity, which is influenced by the interaction of neoplastic cells with the tumour microenvironment. The diagnostic and prognostic role of the tumour stroma in BC remains to be defined. Differential interference contrast (DIC) microscopy is a label‐free imaging technique well suited to visualise weak optical phase objects such as cells and tissue. This study aims to compare stromal collagen fibre characteristics between in situ and invasive breast tumours using DIC microscopy and investigate the prognostic value of collagen parameters in BC. A tissue microarray was generated from 200 cases, comprising ductal carcinoma in situ (DCIS; *n* = 100) and invasive tumours (*n* = 100) with an extra 50 (25 invasive BC and 25 DCIS) cases for validation was utilised. Two sections per case were used: one stained with haematoxylin and eosin (H&E) stain for histological review and one unstained for examination using DIC microscopy. Collagen fibre parameters including orientation angle, fibre alignment, fibre density, fibre width, fibre length and fibre straightness were measured. Collagen fibre density was higher in the stroma of invasive BC (161.68 ± 11.2 fibre/µm^2^) compared to DCIS (*p* < 0.0001). The collagen fibres were thinner (13.78 ± 1.08 µm), straighter (0.96 ± 0.006, on a scale of 0–1), more disorganised (95.07° ± 11.39°) and less aligned (0.20 ± 0.09, on a 0–1 scale) in the invasive BC compared to DCIS (all *p* < 0.0001). A model considering these features was developed that could distinguish between DCIS and invasive tumours with 94% accuracy. There were strong correlations between fibre characteristics and clinicopathological parameters in both groups. A statistically significant association between fibre characteristics and patients’ outcomes (breast cancer specific survival, and recurrence free survival) was observed in the invasive group but not in DCIS. Although invasive BC and DCIS were both associated with stromal reaction, the structural features of collagen fibres were significantly different in the two disease stages. Analysis of the stroma fibre characteristics in the preoperative core biopsy specimen may help to differentiate pure DCIS from those associated with invasion.

## LAY DESCRIPTION

*Lay description*: Breast cancer (BC) is known for its complexity due to the variety of cancer cells and how they interact with the surrounding environment. It is still not fully understood how the surrounding environment affects the BC behaviour. This study explores how the structure of fibres in the surrounding tissue differs between noninvasive and invasive lesions and whether these differences can help predict patient outcomes using specific microscopy called Differential interference contrast (DIC) microscopy. This microscopy allows seeing the tissue without the need for stains.

Methods: This study used tissue samples from 200 BC cases, split evenly between noninvasive and invasive types, plus 50 cases for validation. Each sample was examined in two ways: one was stained for standard examination, and the other was left unstained for a special type of imaging called Differential Interference Contrast (DIC) microscopy. Various features of the collagen fibres were measured, such as their density, thickness, length, straightness, angles and direction.

Findings: The study found that invasive BC tissues had denser, thinner, straighter and more disorganised fibres compared to noninvasive tissues. These differences were significant and could distinguish between noninvasive and invasive cancers with 94% accuracy. Additionally, the characteristics of the collagen fibres were strongly linked to clinical outcomes in invasive but not in noninvasive cases.

Conclusion: The structural differences in fibres between noninvasive and invasive BC could potentially be used to improve diagnosis and predict patient outcomes. This suggests that analysing collagen fibres in biopsy samples before surgery might help identify cases of noninvasive BC from those associated with invasive lesions and distinguish invasive lesions that mimic noninvasive ones.

## 1 INTRODUCTION

Breast cancer (BC), which is the most commonly diagnosed cancer in women worldwide^1^ is a heterogeneous disease with variable morphologies, and clinical behaviours.^2^ The diversity of BC at the morphological level is determined by assessing particular features mainly based on histological tumour type and grade, which are mostly dependent on the tumour neoplastic cell features.^3^ However, the tumour cell‐centric view of BC does not fully explain its heterogeneity and progression. It is increasingly appreciated that the tumour stroma is an integral part of cancer initiation, growth and progression.^4^ Tumour associated stromal response can serve either as a physical barrier to stop tumour progression or as a facilitator to tumour invasion and progression. During cancer progression, the surrounding microenvironment may create a dynamic signalling circuitry that promotes cancer initiation and growth, through continuous paracrine communication with the cancer cells.^4^

It was demonstrated that stromal collagen interacts with tumour cells via cancer cell receptors known as Discoidin domain receptors (DDRs) which promote cell migration through the protein kinase B (AKT) pathway.^5^ Collagen also has a functional ligand, which promotes tumour cell budding and invasion^6^ and acts as a scaffold facilitating the migration of invading tumour cells.^7^ Moreover, collagen organisation and stiffness are affected by crosslinking which results in high tissue tension influencing tumour progression.^8^, ^9^

BC stroma not only influences tumour progression and behaviour and has prognostic significance but also affects diagnosis using clinical, radiological, and pathological methods.^2^ Stromal fibrosis is the main factor responsible for the hard consistency of the tumour making it clinically detectable by palpation and easily detected on imaging.^10^ Stromal elastosis also has a diagnostic role as deposition of elastin material in stroma is associated with early detection in mammography.^11^ On histological examination, tumour associated stromal response, termed as stromal desmoplastic response, is one of the features of malignancy.^11^

The stroma of most BC types is characterised by the deposition of dense fibrous tissue composed of newly synthesised extracellular matrix (ECM) constitutes mainly collagen with or without elastic fibres.^12^ However, the characterisation of BC stroma is a complex and multifactorial challenge. There are several types of stroma observed in BC and distinct combinations of stroma and neoplastic cell features can co‐exist. This makes understanding the role of stroma in BC and its assessment for prognostic analysis challenging.^13^, ^14^, ^15^ Moreover, the desmoplastic stromal reaction is not only observed in invasive BC but also seen in many cases of ductal carcinoma in situ (DCIS), particularly the high nuclear grade. Most of the previous studies reported the prognostic impact of some characteristics of collagen fibre in BC^8^, ^9^; however, not all the fibre characteristics were investigated. In addition, the role of collagen fibre to differentiate between invasive and noninvasive lesions is not covered. The difference between invasive and DCIS stromal ultrastructure features especially the collagen fibre characteristics and their impact on tumour behaviour remains to be defined. Differentiating DCIS from invasive lesions using stromal features is clinically important, particularly in the preoperative core biopsy specimens.^16^ To this end, the use of advanced microscopic techniques can be instrumental in dissecting these morphological characteristics and shed light on stroma features in different disease states.

Differential interference contrast (DIC) microscopy is a label‐free imaging technique well suited to the visualisation of weak optical phase objects such as cells and tissue. Contrast is generated by variations in the sample's optical path (combination of refractive index and physical path length) that alter the extent of light interference between orthogonally polarised beams of light, brightening or darkening the resulting image. DIC microscopy characteristically produces pseudo‐three‐dimensional relief shading, or shadow relief appearance, and has become an extensively used label‐free method to visualise fibrillar structures as it is inexpensive and can be superimposed on other techniques when studying fibrils without the need for staining.^17^

Therefore, to understand the tumour stroma and the differences between DCIS and invasive BC stroma fibres, we investigated the collagen fibre characteristics, the most predominant constituents in the stroma, using DIC microscopy, which has the advantage of visualising collagen fibres without staining. This study aimed to use DIC to identify distinguishing characteristic features of stromal collagen fibres in DCIS and invasive carcinoma and assess the clinical and prognostic value of stromal collagen fibres in BC.

## 2 MATERIALS AND METHODS

### 2.1 Specimens, preparation and staining

A tissue microarray (TMA) containing 200 cores from primary operable BC (pure DCIS, *n* = 100 and invasive BC, *n* = 100) patients who presented to Nottingham City Hospital between 1999 and 2006 was used in this study. Model validation was performed on an additional 50 cases including DCIS (*n* = 25) and invasive (*n* = 25) BC. This study was approved by the Yorkshire & the Humber – Leeds East Research Ethics Committee (REC Reference: 19/YH/0293) and is in line with the Declaration of Helsinki for research.

The TMA construction process started with the retrieval of a representative formalin‐fixed paraffin‐embedded (FFPE) tumour block (donor) for each. A fresh full‐face section of 4 µm thickness was cut from each donor block and stained with H&E and each slide was further reviewed by the pathologist using light microscopy to confirm the presence of the tumour and to identify the most representative areas suitable for array sampling. In cases where the block did not contain adequate tumour tissue for sampling, an alternative tumour block was retrieved and used. TMA cores were marked using Panoramic Viewer Software v. 1.15.4 (3D HISTECH®, Budapest, Hungary), then 0.6 mm tissue core diameter was annotated from the centre and invasion fronts of the tumour, and 4 to 12 cores were marked for each image and one was used for the core. TMA construction was achieved using ordinary paraffin wax‐based blocks prepared by pouring paraffin into blocks (5−10 mm deep) and then extruding the tissue array cores into the recipient block. The TMA blocks were also checked to ensure that they were covered completely but did not have excess wax at the edges or back. Then, the block was trimmed smooth to ensure a flat surface using a microtome.^18^ TMA Grand Master 2.4‐UG‐EN software (3D HISTECH®, Budapest, Hungary) was used to design the block layouts (Supplementary Data 1).

The samples were selected randomly, including all cases of invasive breast cancer or pure DCIS that had available tumour blocks in the archive and their TMA cores were acceptable with adequate amount of tumour cell and tumour‐associated stroma. Exclusion criteria were the cores that had any missed parts had normal breast tissue or did not have any invasive neoplasm. About 65 cores were excluded from the study based on the exclusion criteria. In addition, cases with DCIS with invasive tumours were excluded. Full and detailed clinicopathological data were available (Table 1).

**TABLE 1:** Clinicopathological characteristics of the two study cohorts DCIS and invasive BC.

Parameters	DCIS cohort cases *N* (%)	Invasive cohort cases *N* (%)
Patient age		
≤50 years	32 (32%)	28 (28%)
>50 years	68 (68%)	72 (72%)
Size		
≤2 cm	29 (29%)	65 (65%)
>2 cm	71 (71%)	35 (35%)
Estrogen receptor		
Positive (≥1%)	75 (75%)	82 (82%)
Negative (<1%)	15 (15%)	18 (18%)
Missing	10 (10%)	0 (0%)
Progesterone receptor		
Positive (≥1%)	61(61%)	53 (53%)
Negative (<1%)	29 (29%)	46 (46%)
Missing	10 (10%)	1 (1%)
HER2 status		
Positive (+2, +3)	21 (21%)	21 (21%)
Negative (0,+1)	79 (79%)	79 (79%)
Ki67 score		
High (≥14%)	14 (14%)	43 (43%)
Low (<14%)	75 (75%)	38 (38%)
Missing	11 (11%)	19 (19%)
Molecular subtypes		
Luminal A	63 (63%)	34 (34%)
Luminal B	7 (7%)	38 (38%)
Her2 enriched	5 (5%)	7 (7%)
Triple negative	9 (9%)	10 (10%)
Tumour Grade		
Grade 1/low grade	51 (16%)	18 (18%)
Grade 2/intermediate grade	33 (33%)	37 (37%)
Grade 3/high grade	51 (51%)	45 (45%)
Histological type		
ductal NST		66 (66%)
Lobular		6 (6%)
Special types		28 (28%)
Lymph vascular invasion (LVI)		
Definite		35 (35%)
Negative		65 (65%)

*Note*: This is the clinicopathological data of the study cohort excluding the validation cohort. The table describes the percentage of cases according to different clinicopathological parameters.

Hormone receptor and human epidermal growth receptor 2 (HER2) scoring was assessed according to the UK guidelines and American Society of Clinical Oncology and College of American Pathologists (ASCO CAP) guidelines. Oestrogen receptor (ER) and progesterone receptor (PR) ≥1% were considered positive, while HER2‐positive status was defined as an immunohistochemical (IHC) score of 3+ or 2+ with evidence of gene amplification of HER2 using in situ hybridisation. Ki67 index data was available in the Nottingham cohort, and Ki67 > 14% was classified as a high index.^19^, ^20^

**FIGURE 1:**
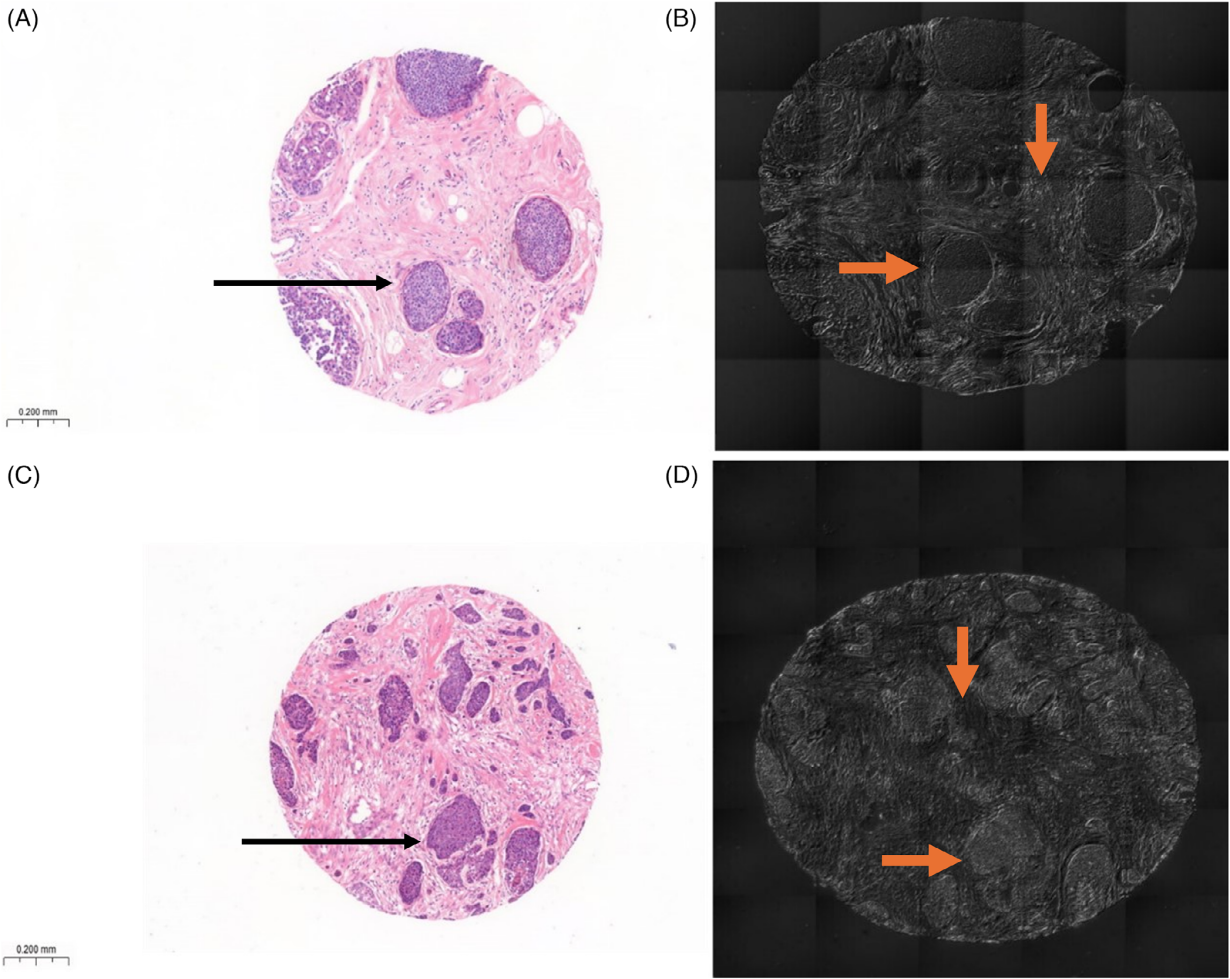
Tissue microarray cores of ductal carcinoma in situ (DCIS) and invasive breast cancer tissue. (A) Haematoxylin & eosin (H&E) stained DCIS core showed DCIS (arrow). (B) Unstained DCIS section examined using differential interference contrast (DIC) microscopy showing DCIS (horizontal arrow) with surrounding stromal fibre features (vertical arrow). (C) H&E‐stained invasive breast cancer core containing neoplastic cells (arrow) invading the stroma. (D) Unstained invasive breast carcinoma section examined by DIC microscopy showing neoplastic cells (horizontal arrow) and the intervening stromal between the invasive cell clusters (vertical arrow). Magnification ×40, scale bar represents 200 µm.

**FIGURE 2:**
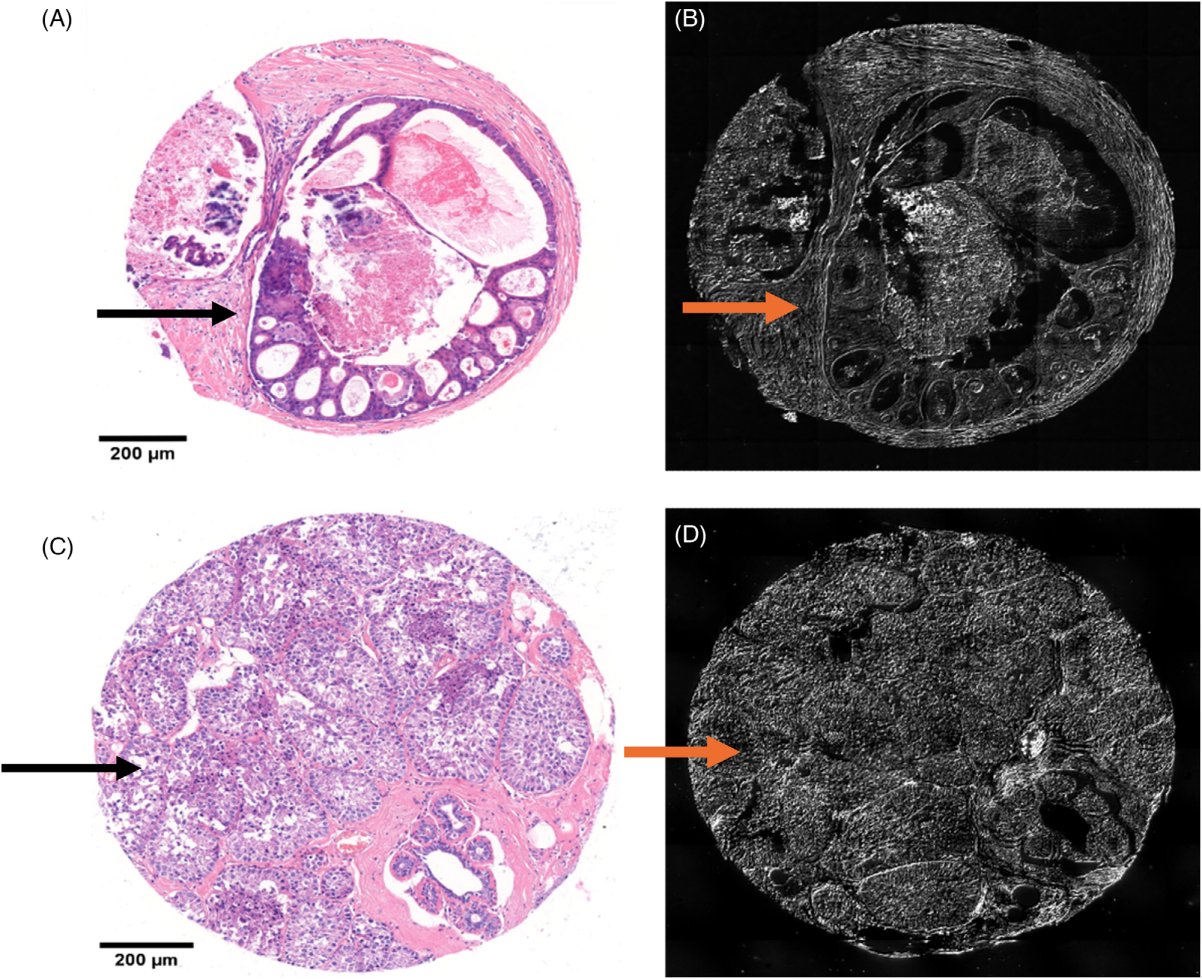
Tissue microarray cores of ductal carcinoma in situ (DCIS) and invasive breast cancer tissue with less stromal tissue and using higher zoom. (A) Haematoxylin & eosin (H&E) stained DCIS core showed DCIS (arrow). (B) Unstained DCIS section examined using differential interference contrast (DIC) microscopy showing DCIS (red arrow). (C) H&E‐stained invasive breast cancer core containing neoplastic cells (arrow). (D) Unstained invasive breast carcinoma section examined by DIC microscopy showing neoplastic cells (red arrow). Magnification ×40, scale bar represents 200 µm.

Two consecutively 4 µm sections per case were prepared from each TMA block: one was stained with haematoxylin and eosin (H&E) stains for histological review, and the second unstained section was examined using DIC microscopy. To this end an Olympus IX83 inverted epifluorescent microscope fitted with a Marzhauser Scan IM 120 × 80 automated scanning stage was used for imaging, using a 20×/0.75 NA objective (Olympus UPlanSApo) together with a c‐mounted 2× amplifying lens (Cairn Research) provided a total magnification of 40×. A sCMOS camera (Photometrics Prime 95b) was used for detection, providing a maximum field‐of‐view of 200 µm × 200 µm. Image acquisition (microscope stage and illumination) was synchronised using an Olympus Real‐Time Controller (RTC) via Olympus CellSens software (Tokyo, Japan). Regions of interest (ROIs) were selected within each core at random from two nonoverlapping areas: one centrally and the other peripherally, each measuring 64 × 64 pixels, the mean value for the two ROIs of each parameter was calculated (Figures 1 and 2).

Outcome data in the form of BC‐specific survival (BCSS), defined as the time from the initial surgical diagnosis to death related to BC, and distant metastasis‐free survival (DMFS), defined as the time from the initial surgical diagnosis to the event of distant metastasis (DM), were collected. The time from surgery to local recurrence is defined as breast or chest wall recurrence while regional recurrence is defined as regional lymph node recurrences, and these were calculated as local recurrence free survival (LRFS) or regional recurrence free survival (RRFS) respectively.

### 2.2 Assessment of collagen fibre features

**FIGURE 3:**
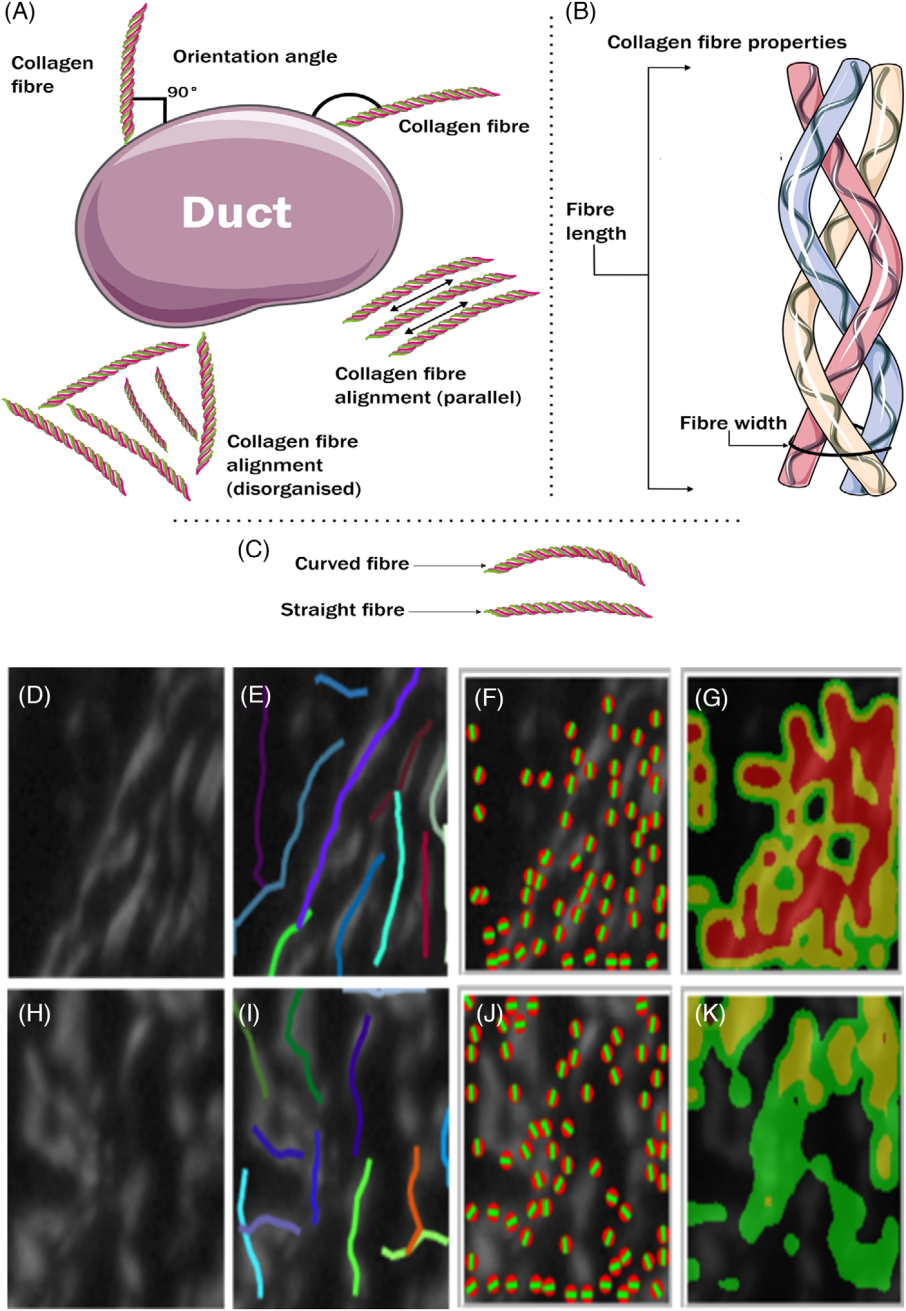
Schematic depicting collagen parameter definitions and measurements. (A) Graph illustrates the orientation angle which is the angle between collagen fibres and duct border which is perpendicular in right angle (90°) or parallel; in addition, it defined the coefficient alignment as the direction of the fibres to each other either good alignment as parallel to each other or disorganised. (B) Graphic describes the characteristics of individual fibres which is formed of three chains including the fibre length which is the end‐to‐end measurement, fibre width which is the fibre diameter and straightness which is the curvature degree of the fibre. (C) Graph describes the fibre straightness and curved fibre. (D, H) Region of interest (ROI) area in either ductal carcinoma in situ (DCIS) stroma (D) or invasive stroma (H). (E, I) The same ROI after analysis using CT‐fire shows highlighted extracted fibre in several colours to measure width, length, and straightness. (F, J) The same ROI analysed with curve align, the image shows red dots which indicate the fibre centre and green lines which indicate fibre orientation. (G, K) A heat map of ROI shows red indicating a well‐aligned region, while yellow indicates moderately aligned areas and green indicates poorly aligned areas. (H) ROI in the stroma of invasive breast cancer.

To extract data from individual fibres, CT‐FIRE (ctFIRE_V2.0Beta_WIN64_MCR2014b.exe) was used to measure the following parameters (measured in pixels and where applicable converted to micrometre, 1 pixel = 2 µm): Mean fibre length (defined as end‐to‐end length of the fibre), mean straightness (defined as the rate of curvature ranged from 0 more curved to 1 straighter), and mean fibre width (Figure 3).

Curve Align (CurveAlign_V4.0Beta_WIN64_MCR2014b.exe) was used to measure the fibre alignment coefficient, which assessed fibre alignment which is the fibre direction compared to each other (unit of measurement ranged from 0 random alignment through to 1 perfect alignment), and the mean orientation angle of fibres compared to a horizontal line resembling the duct border (0°−180° scale; Figure 3). Collagen fibre density (the concentration of collagen fibres in a given area), was assessed via Image J (NIH, Bethesda, Maryland). Density was measured using images at 12‐bits and a macro plugin in Image J

### 2.3 Definitions of the fibre measurements and cut‐offs

Cut‐off values for collagen characteristics, including dimensions and orientations, were generated in X‐tile bioinformatics software (Yale University, version 3.6.1). Cut‐off values were defined for each parameter to specify each parameter into two opposing categories that may be used in clinical settings. Cut‐offs therefore categorised each parameter into two groups for comparison. Cut‐offs for each cohort are shown in Table 2.

**TABLE 2:** The cut‐offs of collagen parameters in the cohorts.

Collagen parameter		DCIS cohort	Invasive cohort
**Orientation angle (degrees)**	**Narrow**	<85.6	<92.9
	**Wide**	>85.6	>92.9
**Fibre alignment (on scale 0–1)**	**Poor**	<0.4	<0.2
	**Good**	>0.4	>0.2
**Fibre width (µm)**	**Thin**	<13.4	<12
	**Thick**	>13.4	>12
**Fibre length (µm)**	**Short**	<132	<122.4
	**Long**	>132	>122.4
**Fibre straightness (on scale 0–1)**	**Curved**	<0.92	<0.96
	**Straight**	>0.92	>0.96
**Fibre density (fibre/µm^2^)**	**Low**	<135.4	<160.6
	**High**	>135.4	>160.6

*Note*: The mean orientation angle means angle between fibre and horizontal line resembling the duct border; fibre alignment means the fibre direction compared to each other; mean fibre length is defined as the end‐to‐end length of the fibre; mean straightness is defined as the rate of curvature ranged from 0 more curve to 1 straighter; and fibre density means the concentration of collagen fibres in a given area.

### 2.4 Statistical analysis

Statistical analyses were performed on the collagen fibre features in SPSS v27 (Chicago, IL, USA) using the Shapiro–Wilk test to check variables distribution and all parameters were normally distributed. Independent *t*‐tests were used to compare collagen characteristics in DCIS to those of invasive tumour lesions. Clinicopathological data was categorised as mentioned in Table 1. Associations between collagen features and clinicopathological parameters were analysed using chi‐square. Univariate survival analysis was completed using a log‐rank test and Kaplan−Meier survival estimates. The Cox regression model was used for multivariate analysis. GraphPad Prism was used to create graphs and undertake these statistical tests analysis.^21^ *p* < 0.05 was considered statistically significant.

A parametric regression model was developed in SPSS using binary logistic regression to predict whether the case is invasive or not (DCIS) based on stromal fibre characteristics. The regression coefficients of the model were estimated to optimise the ability of the model to differentiate between DCIS and invasive tumour orientation angle, alignment, fibre width and fibre density (Table 3). The Omnibus test for model coefficient was significant in all the steps (*p* < 0.001, Table 4). Nagelkerke *R* ^2^ was 0.928 and Cox and Snell *R* ^2^ was 0.696 which indicates a good fit of the model. The goodness of fit Hosmer and Lemeshow test showed that the chi‐square value was 1.23 with a significance of 0.996 which indicates a good fit model. A large value of chi‐squared with *p*‐value < 0.05 indicates poor fit while a small chi‐squared value (with *p*‐value closer to 1) indicates a good logistic regression model fit.

**TABLE 3:** Variables included in the model equation.

					95% Confidence interval for Exp (*B*)	
	Unstandardised coefficient (*B*)	Standard error (SE)	Significance (*p*‐value)	Standardised coefficient Exp (*B*)	Lower	Upper
**Orientation angle (degree)**	0.216	0.068	0.001	1.241	1.087	1.416
**Alignment (scale 0–1)**	−39.505	9.826	0.0001	0.000	.000	.000
**Width (µm)**	−2.276	0.613	0.0001	0.103	.031	.341
**Density (fibres/µm** ^2^)	0.121	0.034	0.0001	1.129	1.056	1.207
**Constant**	3.567	7.058	0.613	35.421		

*Note*: The mean orientation angle means angle between fibre and horizontal line resembling the duct border; fibre alignment means the fibre direction compared to each other; and fibre density means the concentration of collagen fibres in a given area.

**TABLE 4:** Omnibus tests of model coefficients for the model of collagen fibre characteristics to differentiate between DCIS and invasive BC.

		Chi‐square	Degree of freedom (df)	Significance (*p*‐value)
Step 1	Step	236.879	5	0.0001
	Block	236.879	5	0.0001
	Model	236.879	5	0.0001

*Note*: DCIS means ductal carcinoma in situ, BC means breast cancer.

## 3 RESULTS

The mean patient follow‐up in the DCIS cohort was 146 months and the number of cases with recurrence was 15 cases; in the invasive cohort, the mean follow‐up was 372 months and the number of cases that died was 21. The endpoint in the DCIS cohort was the recurrence which is defined as the time from surgery to the recurrence of DCIS while the endpoint of the invasive cohort was BCSS defined as the time from the initial surgical diagnosis to death related to BC.

### 3.1 Collagen geometric characteristics in DCIS and invasive BC

**FIGURE 4:**
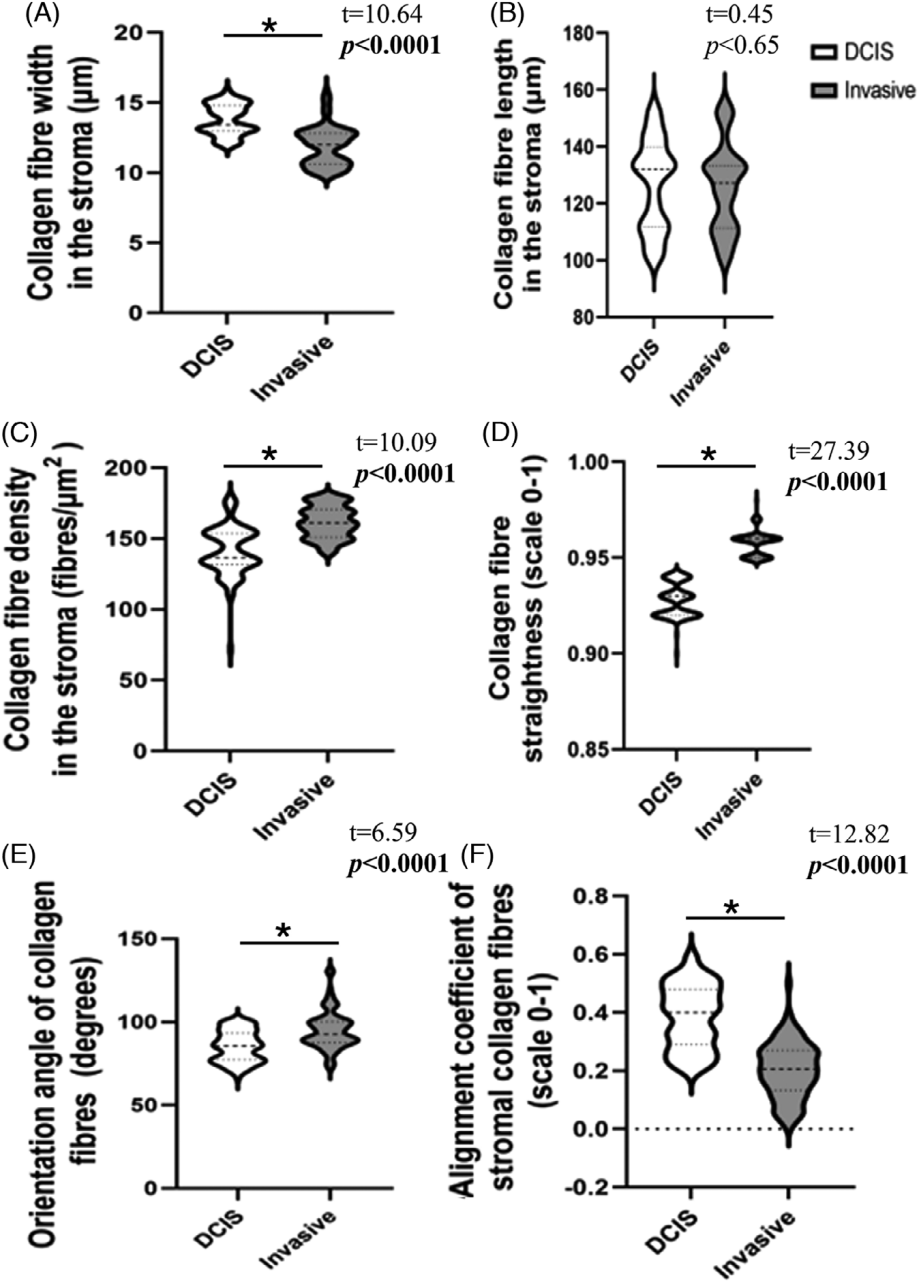
Geometric features of stromal collagen fibres in the DCIS and invasive tissues. (A) Fibre width. (B) Fibre length. C) Fibre density. (D) Fibre straightness. (E) Fibre orientation angle; (F) fibre alignment. Measurements obtained from unstained TMA sections using differential interference contrast (DIC) microscopy ×40. Two ROIs (32 × 32 µm) for each core, CT‐FIRE and Curve Align, were used to assess measurements, *N* = 100 cores/group. Independent *t*‐test. DCIS = ductal carcinoma in situ, invasive = invasive breast carcinoma.

Comparisons of the stromal collagen fibre characteristics between the DCIS and invasive BC groups showed a significantly lower fibre width and higher mean collagen fibre density (*t* = 10.64, independent *t*‐test, *t* = −10.09, respectively *p* = 0.0001; Tables 5 and S1) in the invasive group compared to DCIS. The invasive tumour stroma showed straighter collagen fibres than those in the stroma surrounding DCIS ducts (independent *t*‐test, *t* = −27.39; *p* = 0.0001). In contrast, there was no significant difference in stromal collagen fibre lengths between the DCIS and invasive groups (independent *t*‐test = 0.65; *p* = 0.45; Figure 4, Tables 5 and S1).

**TABLE 5:** Comparisons of the stromal collagen fibre geometric characteristics in the invasive and DCIS cohorts showing the comparison in the means value of each collagen parameter.

Stromal collagen fibre characteristics	DCIS (*N* = 100) (mean ± SD)	Invasive (*N* = 100) (mean ± SD)	*p* Value (*significance *p* < 0.05)
Mean fibre width (µm)	13.78 ± 1.08	11.9 ± 1.38	0.0001
Mean fibre length (µm)	126.8 ± 15.36	125.8 ± 15.18	0.45
Mean fibre density (fibre/µm^2^)	141.22 ± 17.18	161.68 ± 11.2	0.0001*
Mean fibre straightness (0–1 scale)	0.93 ± 0.009	0.96±0.006	0.0001*
Mean orientation angle (degree)	85.40 ± 9.26	95.07±11.39	0.0001*
Alignment coefficient of fibres (0–1 scale)	0.39 ± 0.11	0.20±0.09	0.0001*

*Note*: DCIS = ductal carcinoma in situ, Invasive = invasive breast carcinoma, independent *t*‐test used to assess statistical significance. Fibre straightness ranged between 0.90 and 0.93 in the DCIS cohort while it ranged from 0.95 to 0.98 in the invasive group producing a significant difference between the cohorts. Mean fibre length is defined as the end‐to‐end length of the fibre; fibre density means the concentration of collagen fibres in a given area; mean straightness is defined as the rate of curvature ranged from 0 more curve to 1 straighter; the mean orientation angle means angle between fibre and horizontal line resembling the duct border; and fibre alignment means the fibre direction compared to each other.

*p < 0.05.

Concerning the direction of the stromal collagen fibre orientations, the fibres within the invasive carcinoma stroma were significantly more perpendicular, as measured against the duct border (95.07° ± 11.39°) compared to those in the DCIS stroma (85.40° ± 9.26°; independent *t*‐test, *t* = −6.59; *p* = 0.0001; Tables 5 and S1). When comparing the fibres within each sample to each other, the DCIS stromal fibres were more parallel and exhibited organised fibre alignment (0.39 ± 0.11 on a 0–1 scale) compared to the fibres observed in the invasive stroma (0.20 ± 0.09 on a 0–1 scale independent *t*‐test; *t* = 12.82; *p* = 0.0001; Tables 5 and S1, Figure 4).

### 3.2 Model to differentiate between DCIS and invasive tumours

For clinical application, a model to predict whether any case is an invasive lesion or not invasive was performed based on these stromal fibre characteristics which showed significant differences between both cohorts.

The equation would be calculated as PInvasive = (3.567) + (0.216 × orientation angel) + ((−39.505) × alignment) + ((−2.276) × width) + (0.121 × density) if the PInvasive score was less than 0.5, so the case would be DCIS, whereas in cases more than 0.5, it would be an invasive tumour.

**FIGURE 5:**
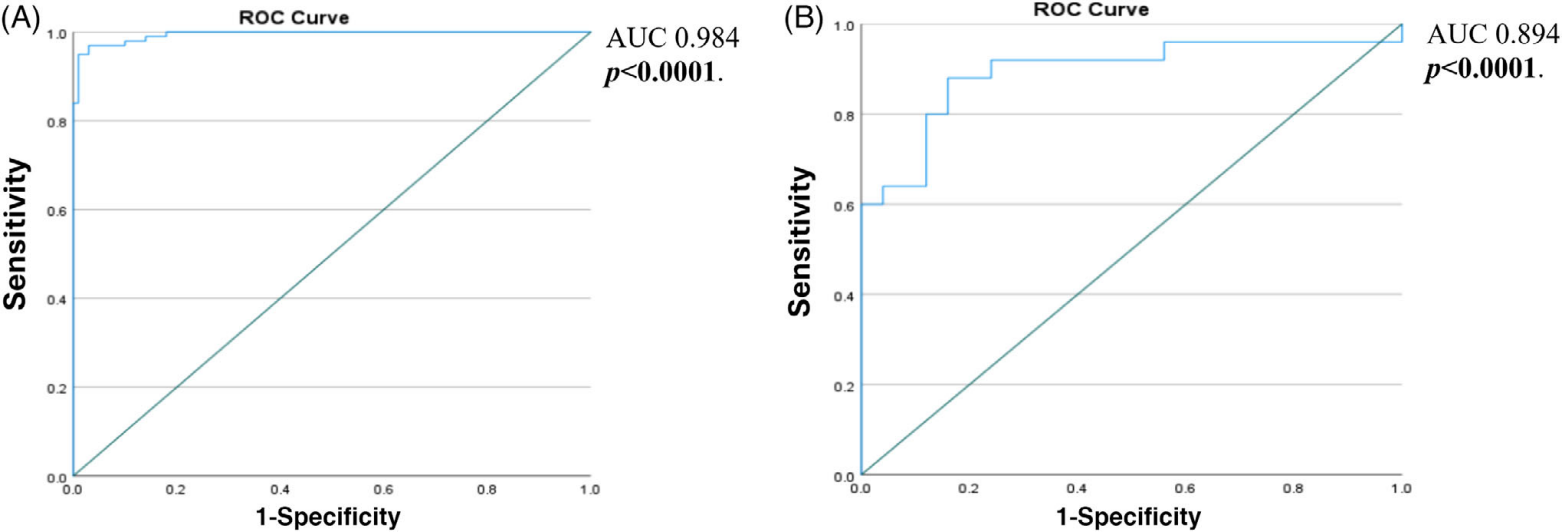
Receiver operating characteristic (ROC) curve showed model performance. (A) Internal validation of the training cohort. (B) External validation of validation cohort.

To test the model, performance analysis based on receiver operating characteristic (ROC) was used. The area under the curve (AUC) was 0.984 (*p* = 0.0001) with sensitivity 98% and specificity 95% according to the AUC table in SPSS, which indicated good discrimination of the model (Figure 5A).

A classification matrix table was calculated to measure the model performance (Table 6).

**TABLE 6:** Classification matrix for sensitivity and specificity of the collagen characteristic model for the main cohort.

		Predicted		Percentage of true predicted
		DCIS	Invasive	
Observed	DCIS (*N* = 100)	97 (TN)	3 (FP)	97.0
	Invasive (*N* = 100)	3 (FN)	97(TP)	97.0
Overall percentage				97.0
Sensitivity		(TP)/(TP + FN) = 97/(97 + 3) = 97%		
Specificity		(TN)/(TN + FP) = 97/(97 + 3) = 97%		
Accuracy		TP + TN/TP + TN + FP + FN = 97 + 97/200 = 97%		
Precision		TP/(TP + FP) = 97%		

*Note*: The cut value is 0.500. DCIS = ductal carcinoma in situ. TN = true negative, FP = false positive, FN = false negative, TP = true positive. TP = number of cases had probability to be invasive and were invasive. TN = number of cases had probability to be DCIS and were DCIS. FP = number of cases had probability to be invasive but were DCIS. FN = number of cases had probability to be DCIS but were invasive.

An independent (validation) cohort (50 cases) was analysed for collagen fibre features, then the estimated equation was used to predict their type. ROC was performed to measure the performance of the model and AUC was 0.894 (*p* = 0.001) which indicates good discrimination (Figure 5B).

A classification matrix table was formed to measure the model performance. Sensitivity was 96% while specificity was 92%, accuracy was 94% and precision was 96% (Table 7).

**TABLE 7:** Classification matrix for sensitivity and specificity of the collagen fibre characteristics model for the validation cohort.

		Predicted		Percentage of true predicted
		DCIS	Invasive	
Observed	DCIS (*N* = 50)	23 (TN)	2 (FP)	92.0
	Invasive (*N* = 50)	1 (FN)	24 (TP)	96.0
Overall percentage				94.0
Sensitivity		(TP)/(TP + FN) = 24/(24 + 1) = 96%		
Specificity		(TN)/(TN + FP) = 23/(23+2) = 92%		
Accuracy		TP + TN/TP + TN + FP + FN = 24 + 23/50 = 94%		
Precision		TP/(TP + FP) = 96%		

*Note*: The cut value is 0.500. DCIS = ductal carcinoma in situ. TN = true negative, FP = false positive, FN = false negative, TP = true positive. TP = number of cases had probability to be invasive and were invasive. TN = number of cases had probability to be DCIS and were DCIS. FP = number of cases had probability to be invasive but were DCIS. FN = number of cases had probability to be DCIS but were invasive.

### 3.3 Association of stromal collagen characteristics with clinicopathological data in DCIS

Correlations between collagen features and clinicopathological parameters are presented in Tables S2 and S3.

Specific fibre features including wide orientation angle, poorly aligned, thicker, longer straighter fibres with a higher fibre density, as defined in the materials and methods, were associated with features of aggressive DCIS behaviour (Tables S2 and S3).

Wide orientation angle was associated with features of aggressive DCIS behaviour including higher nuclear grade (*p* < 0.001), DCIS heterogeneity (*p* < 0.02), comedo necrosis (*p* < 0.02) TNBC subtype (*p* < 0.001), hormone receptor negativity (*p* < 0.001), high proliferation status HER2 positivity (*p* < 0.03) and (high ki67 score; *p* < 0.001) (Table S2).

Poorly aligned fibres were associated with aggressive DCIS behaviour including large size (*p* < 0.003), higher nuclear grade (*p* < 0.001), comedo necrosis (*p* < 0.001), TNBC subtype (*p* < 0.001), hormone receptor negativity (*p* < 0.001), and high proliferation status (high ki67 score; *p* < 0.003) (Table S2). In addition, straighter fibres were associated with large size (*p* < 0.001) higher nuclear grade (*p* < 0.001), comedo necrosis (*p* < 0.001), TNBC subtype (*p* < 0.001), hormone receptor negativity (*p* < 0.001), HER2 positivity (*p* < 0.03) and high proliferation status (high ki67 score; *p* < 0.003) (Table S2).

Thick fibres were correlated with large size (*p* < 0.001), higher nuclear grade (*p* < 0.001), comedo necrosis (*p* < 0.001), TNBC subtype (*p* < 0.001), hormone receptor negativity (ER status *p* < 0.002 and PR status *p* < 0.001), HER2 positivity (*p* < 0.04) and high proliferation status (high ki67 score; *p* < 0.001). While longer fibres were associated with large size (*p* < 0.002), higher nuclear grade (*p* < 0.001), heterogeneity (*p* < 0.02), comedo necrosis (*p* < 0.001), TNBC subtype (*p* < 0.002), hormone receptor negativity (*p* < 0.001), HER2 positivity (*p* < 0.03), and high proliferation status (high ki67 score; *p* < 0.04) (Table S3).

High density was associated with large size (*p* < 0.01), higher nuclear grade (*p* < 0.001), comedo necrosis (*p* < 0.001), TNBC subtype (*p* < 0.001), hormone receptor negativity (*p* < 0.001), and high proliferation status (high ki67 score; *p* < 0.004) (Table S3).

### 3.4 Association of stromal collagen characteristics with clinicopathological data in invasive group

Associations between specific stromal collagen parameters and the clinicopathologic data in the invasive BC group were presented in Tables S4 and S5.

In invasive tumours, the orientation angle was associated with histological grade only (*p* < 0.001; Table S4).

Poor fibre alignment was associated with aggressive clinicopathological parameters as higher grade (*p* < 0.001), higher Nottingham prognostic index (NPI) scores (*p* < 0.001), molecular subtypes (*p* < 0.001), morphological subtype (*p* < 0.012), LVI (*p* < 0.002) high stage (*p* < 0.009), ER and PR negativity (*p* < 0.001) (Table S4).

Straightness was associated with aggressive clinicopathological parameters. Straight fibres were correlated with large tumours (*p* < 0.001), worse NPI (*p* < 0.001), LVI (*p* < 0.001), higher grade (*p* < 0.001), histological subtype (*p* < 0.004), molecular type (*p* < 0.001), hormonal receptors negativity, ER (*p* < 0.005) and PR (*p* < 0.014), HER2 positivity (*p* < 0.032) and high ki67 (*p* < 0.001) (Table S4).

Width was associated with aggressive clinicopathological parameters as large tumour size (*p* < 0.004), higher histological grade (*p* < 0.001), higher stage (*P*<0.001), poor NPI (*p* < 0.001), LVI (*p* < 0.035), NST subtype (*p* < 0.001), molecular subtype (*p* < 0.001), hormonal receptors negativity (*p* < 0.001), HER2 positivity (*p* < 0.001) and high ki67 (*p* < 0.001) (Table S5).

Length was correlated with aggressive clinicopathological parameters as short fibres were associated with the worse molecular class (*p* < 0.002), high grade (*p* < 0.001), high stage (*p* < 0.013), LVI (*p* < 0.002), poor NPI (*p* < 0.001), nonspecial type (NST) subtype (*p* < 0.007), ER negativity (*p* < 0.001), PR negativity (*p* < 0.009), HER2+ status (*p* < 0.003) and high ki67 (*p* < 0.001) (Table S5).

High fibre density was associated with some aggressive clinicopathological parameters as large tumours (*p* < 0.004), high grade (*p* < 0.001), high stage (*p* < 0.005), poor NPI (*p* < 0.001), LVI (*p* < 0.004), histological subtype NST (*p* < 0.001), molecular subtypes (*p* < 0.001), ER negativity (*p* < 0.012), HER2 positivity (*p* < 0.003) and high ki67 (*p* < 0.001) (Table S5).

### 3.5 Association of stromal collagen characteristics in invasive cohort with outcome

**FIGURE 6:**
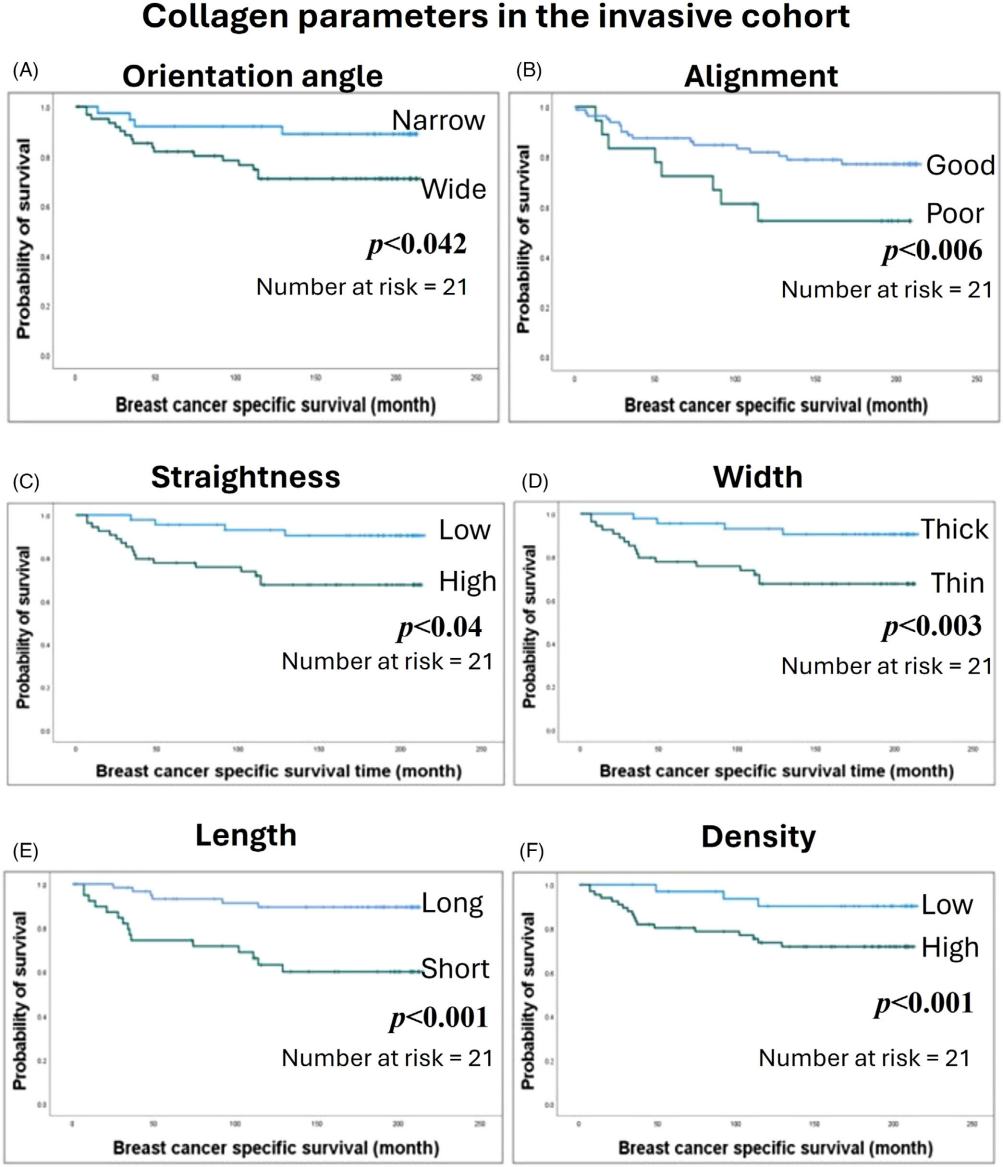
Kaplan–Meier survival plots showing breast cancer‐specific survival (BCSS) of cases with stromal collagen parameters: (A) orientation angle, (B) alignment, (C) straightness, (D) fibre width, (E) fibre length, (F) fibre density.

In invasive BC, a wide fibre orientation angle was associated with shorter breast cancer specific survival (BCSS; HR = 0.17, 95% CI = 0.05 to 0.51, *p* = 0.042; Figure 6A) and shorter RRFS (HR = 0.07, 95% CI = 0 to 0.009, *p* = 0.012; Figure S3A). Patients whose BC stroma showed poor fibre alignment, or had straighter fibres, had shorter BCSS (HR = 3.7, 95% CI = 1.35 to 10.13, *p* = 0.006 and HR = 0.29, 95% CI = 0.08 to 0.98, *p* = 0.04 respectively; Figure 6B and C, respectively). In addition, poor alignment fibres showed shorter DMFS (*p* < 0.03; Figure S3B) while straightness showed no association with other patient outcomes (Figures S1C, S2C and S3C). Similarly, stroma with thicker collagen fibres (Figure 6D) or shorter fibres (Figure 6E) was associated with shorter BCSS (HR = 0.24, 95% CI = 0.09 to 0.62, *p* = 0.003, and HR = 4.64, 95% CI = 1.79 to 11.97, *p* = 0.001 respectively) and shorter DMFS (HR = 0.17, 95% CI = 0.06 to 0.45, *p* < 0.029; HR = 4.64, 95% CI = 1.79 to 11.97, *p* < 0.06 respectively; Figure S3D and E regularly). Higher fibre density was also correlated with shorter BCSS (HR = 0.045, 95% CI = 0.006 to 0.334, *p* = 0.001), shorter RRFS (HR = 0.092, 95% CI = 0.012 to 0.730, *p* = 0.06) and shorter DMFS (HR = 0.069, 95% CI = 0.016 to 0.259, *p* = 0.01; Figures 6F, S2F and S3F).

Multivariate analyses revealed that the fibre alignment, fibre density, fibre length and fibre width were independent prognostic indicators for BCSS (*p* = 0.03, *p* = 0.003, *p* = 0.01 and *p* = 0.002, respectively) independent of tumour size, LVI, lymph node status and molecular classes. Regarding molecular subclasses, in luminal A, fibre width (*p* = 0.02) and fibre straightness (*p* = 0.001) were an independent prognostic indicator for BCSS while in TNBC, fibre alignment (*p* = 0.04) was an independent prognostic indicator for BCSS tumour size, LVI and lymph node status.

There were no associations between any of the fibre parameters (orientation angle, alignment, width, length, straightness, or density) and patients’ outcomes in the DCIS cohort were identified (Supplementary Data 2).

## 4 DISCUSSION

The observations of the present study show that the collagen fibre density was higher in invasive breast cancer (BC) compared to DCIS stroma. The collagen fibres were thinner, straighter, more perpendicular, more disorganised and less aligned to each other in the invasive BC group compared to DCIS. Despite these morphological differences, collagen fibre length was not statistically different between the two groups. These results support previous reports by Lee and colleagues^22^ that progressive changes in stromal collagen organisation play a key role in tumour progression and malignancy by promoting a tumour‐permissive microenvironment, especially for invasive BC.

Different cut‐offs were used for collagen characteristics depending on the patient outcomes for each group separately, which gave different values for each group to define the collagen parameters in each group due to two different values for each cohort and different patient outcomes not depending on the median. In the DCIS cohort based on local recurrence‐free survival (LRFS) while in an invasive cohort based on breast cancer free survival (BCSS), the cut‐off values related to the outcome. The cut‐off divides each parameter into two groups to compare between them for example collagen fibre length to short and tall fibres. This reduces potential bias that may have existed if the same cutoff had been used for both groups or the mean was used depending on the various measures.^23^

Regards to fibre orientation and alignment, the observations in the present study showed that the fibres were more perpendicular to the duct border in invasive carcinoma stroma. These results are consistent with a previous study, which defined morphological modifications in the arrangement and deposition of the collagen fibres which are known as Tumour‐Associated Collagen Signatures (TACs) showing that collagen fibres with a higher perpendicular orientation angle (TACS‐3) resulted in building highways for tumour cells migration.^24^ Another study assessing collagen fibres in human luminal BC was measured using second‐harmonic generation microscopy (SHG) and analysed with Image J. This previous study reported that stromal disorganisation and uniformity correlated with poor patient outcomes.^25^ This finding was consistent with the present study results showing a correlation between the wide orientation angle (90°) with worse BC outcomes (*p* = 0.042), in addition to the association between poor alignment fibres with worse BC outcomes (*p* = 0.001). It was hypothesised that this radial orientation of collagen fibres resulted from contractile and morphogenic processes from tumour boundaries, to reorganise the extracellular matrix (ECM) for local invasion and metastasis.

In normal breast tissue, stromal collagen fibres are wavy and anisotropic; however, in neoplastic tissue, they show progressive thickening and straightness of the fibres.^26^ However, DCIS and benign tumours show a mixed collagen fibre similar to both normal and malignant collagen fibres.^27^ Regarding straightness, we showed that collagen fibres were straighter in the invasive groups compared to DCIS, where the fibres were more curved. These results are consistent with a study by Arwert and colleagues which reported that tumour cells migrated along straighter, unidirectional, collagen fibres towards blood vessels during invasion.^28^ In addition, realignment and/or straightening of these collagen fibres has been correlated with local invasion, metastasis, and poor patient outcomes in breast cancer.^29^ The present study showed consistent results as there were significant correlations between poor fibre alignment and straightness of the fibres with worse patient outcomes. Straightening of the collagen fibres could result in ECM porosity changes which enhance the cell's requirements to leave the tumour by invading adjacent tissues.^30^

Concerning fibre density, we also showed that collagen density was greater in the invasive group compared to DCIS, this aligned with previously published work evaluating ECM stiffness in normal, benign, and invasive breast cancer. The previous work revealed that breast cancer aggressiveness was associated with an incremental increase in collagen deposition and density.^31^ Many studies have shown that increased collagen density was associated with tumour metastasis and poor patient outcomes in human breast cancer^32^, ^33^, ^34^, ^35^ which was consistent with our results as high density was associated with worse patient outcomes.

Indeed papillary, mucinous, and medullary BC tumours, which generally exhibit indolent clinicopathologic behaviours, have been shown to exhibit distinct collagen fibre characteristics as compared to more aggressive invasive tubular and lobular carcinomas,^36^, ^37^ consistent with results reported here which confirm an association between low‐density, organised, well‐aligned fibres with the special histological subtype (Tables S4 and S5). Moreover, this is in agreement with other studies which used various types of microscopies (polarised, SHG, two‐photon, multiphoton and atomic force microscopy (AFM)) to compare the collagen architecture in different molecular subtypes which showed that highly aggressive BC subtypes (including TNBC, HER2 positive and luminal B) had higher collagen density, thicker and straighter compared to less aggressive subtypes^38^, ^39^ which is consistent with our results.

Regards fibre width and length, it has been previously reported that fibre length was a significant prognostic factor for overall survival and disease free survival in a TNBC cohort.^40^ Kullage and colleagues reported a correlation between tumour differentiation and collagen fibre width, where well‐differentiated tumours were associated with thicker fibres, and poorly differentiated tumours harboured thinner collagen fibres.^41^ This is consistent with our results that showed thinner collagen fibres were associated with invasive tumours.

Although the comparison of fibre characteristics between DCIS and the invasive cohort group showed significant differences in all the parameters except fibre length, the association between fibre characteristics and patients outcomes in both groups was different. This may be explained by the following statements: (1) the different circumstances of the disease nature; (2) the clinical events in the DCIS group are rare which may affect outcome analysis in this cohort; and (3) The role of stroma is different in DCIS and invasive tumours. In DCIS tumour cells are separated from the surrounding stroma by a basement membrane and a layer of myoepithelial cells while in invasive tumour the tumour cells are in direct contact with the surrounding stromal elements and direct communication and interplay are more likely to occur. In DCIS, the intraductal tumour cells at the areas of potential invasion may change the adjacent stroma structure and composition to be prepared for such process through paracrine or direct effect and surrounding stroma may facilitate or prevent such early invasion process. In invasive tumours, the stroma plays a major role not only in the motility of the invasive tumour cell but also in the progression and metastasis through several mechanisms including interplay and cross between both components providing a suitable microenvironment for the tumour to acquire more or less invasiveness and aggressive features.^8^, ^9^, ^42^

DIC microscopy is a relatively easy technique that does not require tissue staining that could potentially highlight collagen features with the aid of artificial intelligence (AI) which could become a simple technique to use in practice. The power of DIC microscopy is that it does not require extensive specimen processing such as fixation, dehydration and staining which entails a risk of artefact in each step that can lead to underestimation or overestimation of analysis.^17^ Although AFM has the same advantage, the irregular surfaces affect its resolution. For example, high interfibrillar collagen porosity does not allow individual collagen fibrils to be imaged and needs special sectioning and surface‐finishing techniques,^43^ this is in contrast to the previous work using SHG microscopy^44^ which gives strong signals for collagen I and II but does not detect collagen IV and V.^45^ However, SHG microscopy is an expensive method with less accuracy and some limitations as it is commonly used for unipolar fibrils with constant collagen fibre density. The signal of SHG also depends on the angles of the fibres which may lead to inaccuracies in quantitative studies,^46^ especially given the differing angles observed in the present study. Multiphoton microscopy also has the same advantages but is limited to the resolution and diffraction limit. A super‐resolution detector needs to image collagen fibres that surpass the diffraction, reflecting a complex process.^47^

In routine pathological analysis, immunohistochemical staining for myoepithelial cells (MEC) has been used for a long time in diagnostic procedures when differentiating between noninvasive and invasive BC. However, in some benign lesions, the MEC layer may be absent in noninfiltrative cases such as apocrine lesions and some benign BC such as micro glandular adenosis and infiltrating epitheliosis. In addition, 20% of radical sclerosing cases showed absent MECs.^48^

In this study, a diagnostic model to differentiate in situ (DCIS) from invasive BC was developed based on the most significantly different stromal fibre characteristics between the two disease stages. This mode provided an accuracy of 97% with high sensitivity and specificity supporting its diagnostic role, particularly in the preoperative core needle biopsy. There is no significant difference between the malignant cells of DCIS and grade‐matched invasive BC cells apart from the demonstration of the myoepithelial cell layer and/or basement membrane at the tumour stroma interface. Most of the parameters showed differences between DCIS and invasive stroma and were associated with outcome; however, we found that we could use these parameters in the model to differentiate between both lesions. The model includes all parameters except the fibre length and straightness as there was no significant difference between DCIS and invasive fibre length however, the significant difference in the straightness is not used in the model. The straightness range in DCIS (0.90−0.94), whereas in the invasive group (0.95−0.98), there was no overlapping between both groups which would give no value to the model, so it was excluded. This study demonstrated the ability of DIC microscopy utilising stromal collagen fibre characteristics to differentiate between DCIS and invasive BC which could enable pathologists to predict which DCIS cases are likely to be associated with invasive carcinoma. However, these results require further validation using a larger number of cases and analysing the stroma around DCIS in both pure DCIS and DCIS associated with invasive disease.

Digital image analysis and machine learning have been used to develop algorithms that could identify stromal alterations in benign and malignant BCs in addition to grading carcinomas in situ based on stromal characteristics to predict invasion and malignancy.^49^ Computational algorithm assessment for collagen distribution is a challenge for the clinical utility of image‐based collagen fibre biomarkers as collagen fibre matrices such as alignment, density, orientation, individual fibre measurements and their relation to tumour gland have diagnostic and prognostic value.^50^ However, Keikhosravi et al. developed a model that extracted these characteristics from H&E sections based on a trained neural network of SHG data, but their clinical value requires more validation.^51^ Therefore, we used DIC microscopy as it visualises collagen fibres without stain which could provide more information that helps further in validation.

These measurements could help differentiate between DCIS and invasive tumours in the clinical setting. In addition, collagen plays a vital role in cancer therapy resistance. For example, high collagen density was associated with chemotherapy resistance in oesophageal cancer^52^ and pancreatic cancer.^53^ Collagen density measurement could help us in management decisions.

The potential limitations of this study are the small sample size and low event rate in the DCIS group. However, this study aimed to find a signature of collagen characteristics that could differentiate between DCIS and invasive BC and its diagnostic application regardless of the prognostic value. The information about the margin distance was not available, but all cases were completely excised. Patients with close or positive margins had re‐excision of the index margin(s) or mastectomy according to local protocols. Therefore, the outcome data for local recurrence are unlikely to be affected by margin status.

In conclusion, the study aimed to ascertain whether distinct stromal collagen fibre features could be related to functional differences in the tumour microenvironment, molecular phenotypes and outcomes in BC. Here distinct collagen fibre features were identified between DCIS and invasive carcinomas. These differences in stromal collagen features could be used as useful diagnostic features in differentiating between in situ and invasive lesions via the use of image analysis except for length which is not significantly different between both groups. Fibre length could not be useful in diagnostics to differentiate DCIS from invasive carcinoma and showed a predictive value in patients with invasive BC.

For invasive BC, fibre density and width could be used as a predictive indicator for BC in addition to the fibre's alignment for BCSS and risk of recurrence. The study highlights the role of fibre length as a predictor factor that could be used in BC. Future work studying the mechanisms behind these collagen signatures could further discriminate between tumour types assist patient outcome predictions and help develop novel therapies.

To summarise, the collagen parameters are predictive of outcomes in the invasive group including recurrence and survival. Collagen fibre characteristics are not predictive of outcome endpoints in DCIS. Moreover, fibre characteristics can differentiate DCIS from invasive diseases in the breast.

## AUTHOR CONTRIBUTIONS

SG examined slides with DIC microscopy, scored the cases with data analysis and interpretation and wrote the manuscript draft. CR and GB helped with data interpretation. TB helped in microscopy examination and manuscript revision. RP assisted in graphic illustration and manuscript revision and approved the final version. SG, CR, CA, MA, MM, GB and NM wrote the article, agreed with the article results, critically reviewed the article and approved the final version. E. Rakha conceived and planned the study, contributed to data interpretation, made critical revisions, and approved the final version.

## CONFLICT OF INTEREST STATEMENT

The authors declare no conflicts of interest.

## ETHICS STATEMENT

This study obtained ethics approval to use the human tissue samples from the Northwest – Greater Manchester Central Research Ethics Committee under the title; Nottingham Health Science Biobank (NHSB), reference number 15/NW/0685. Informed consent was obtained from all individuals and all samples were anonymised.

## Supporting information



Supporting Information

Supporting Information

Supporting Information

Supporting Information

Supporting Information

Supporting Information

Supporting Information

Supporting Information

Supporting Information

Supporting Information

Supporting Information
