# Supplementary material for: Geometric characteristics of stromal collagen fibres in breast cancer using differential interference contrast microscopy
Source: J Microsc. 2024 Oct 3;297(2):135–52. doi: 10.1111/jmi.13361 (PMC11733853; doi:10.1111/jmi.13361)
Supplement: Supplementary file 1 — Supporting Information [file JMI-297-135-s002.docx]

**Supplementary Figure 1.** Kaplan Meier survival plots showing Local recurrence free survival,of cases with collagen parameters. **(A)** Orientation angle, **(B)** Alignment, **(C)** Straightness, **(D)** Fibre width, **(E)** Fibre length, **(F)** Fibre density.

**Supplementary Figure 2.** Kaplan Meier survival plots showing Regional recurrence free survival (RRFS)of cases with collagen parameters. **(A)** Orientation angle, **(B)** Alignment, **(C)** Straightness, **(D)** Fibre width, **(E)** Fibre length, **(F)** Fibre density.

**Supplementary Figure 3.** Kaplan Meier survival plots showing Distant metastasis free survival (DMFS of cases compared to collagen parameters. **(A)** Orientation angle, **(B)** Alignment, **(C)** Straightness, **(D)** Fibre width, **(E)** Fibre length, **(F)** Fibre density.
