# Supplementary material for: Geometric characteristics of stromal collagen fibres in breast cancer using differential interference contrast microscopy
Source: J Microsc. 2024 Oct 3;297(2):135–52. doi: 10.1111/jmi.13361 (PMC11733853; doi:10.1111/jmi.13361)
Supplement: Supplementary file 2 — Supporting Information [file JMI-297-135-s011.docx]

**TMA construction process**

The steps of the TMA construction process are collecting the cohort to be arrayed, preparing the recipient array blocks, designing the array grids, constructing the individual array blocks, and preparing the array block for sectioning.

### **Preparation of the donor blocks**

For proper construction tissue array, sampling the representative site from the donor block is a critical step. To identify the suitable cases that could be used to construct the TMA, previously reviewed slides were selected and scanned.

Formalin-fixed paraffin-embedded (FFPE) tumour block (donor) that was characteristic of the specimens from each patient was taken from the tissue block archives. The blocks included were ≥ 4 mm in depth bearing viable tumour tissue and a suitable tumour burden. The priority was given to the blocks that showed adequate tumour tissue which had not been frequently sectioned for previous studies.

### **Matching of donor blocks with their corresponding slides**

To identify the areas that would be most appropriate for the TMA sampling, the presence of the tumour was confirmed by reviewing the digital slides and comparing them with the retrieved blocks. Visual assessment of blocks against the slides was performed to find matched blocks with slides. To avoid the potential risk of consuming a patient’s diagnostic material, tumours with only one block or small tumour tissue samples were excluded to ensure the diagnostic patient’s materials would not be depleted.

**Digital images marking**

The TMA cores were marked by Panoramic Viewer Software v. 1.15.4 (3D HISTECH®, Budapest, Hungary). TMA cores were selected on the basis of the higher density of invasive tumours. Annotation of 0.6 mm tissue core diameter in the centre of the tumour . About 4 to 12 cores were marked per image to use one after that.

**Recipient block construction**

TMA construction was accomplished using the ordinary paraffin wax-based blocks which had been prepared by pouring the paraffin into blocks about 5–10 mm in depth and then extruding the tissue array cores into the block.

To prepare the recipient blocks, plain molten paraffin wax (56°–58°C) was poured into a mould and then topped with a histopathological tissue cassette. The blocks were cooled on ice until the wax hardened and the wax was checked for the presence of air bubbles, striations or any fault line . The block was checked to ensure that it was covered completely without any excess wax at the edges or back. Finally, the block was trimmed smoothly using a microtome to ensure a flat surface

### **TMA layout design**

TMA Grand Master 2.4-UG-EN software (3D HISTECH®, Budapest, Hungary) was used to design the block layouts. The creation of a new layout followed by a new layout name was inserted. The standard block size of 24x35 mm. Four replications were prepared for each block, each was given a unique version number, that was added to the block.

Each array contained 120 samples arranged in three subarrays, with a 1mm space between each core. The first subarray was composed of the 53 cores (6 rows, 10 columns) in the upper half of the block. The second subarray of the block was made up of 40 cores (4 rows, 10 columns), while the third subarray contained 23 cores (3 rows, 10 columns). In each subarray, cores were arranged at a centred distance, and the subarrays were separated by a single gap for orientation and macroscopic identification of the TMA block. Positive internal controls were introduced by adding orientation cores from the normal kidney, normal liver and tonsil tissues outside of the geometric margin of the array on the top and bottom left-hand sides.

Documentation of both the donor block and the TMA position of each core was done. An array map grid was performed for each array block. Each grid had a unique identifier and was organised in the same sequence corresponding to its position in the array block.

### **TMA block annealing and sectioning**

### After TMA block construction, Blocks were placed face-up in an oven for three hours at 37°C to anneal them helping the tissue cores to adhere to the holes walls in the array block and making the wax flexible for handling.

### A clean glass microscope slide was placed on the face surface of the block, then gentle pressure was applied to push all the cores on the array to the same level. The blocks were left to cool at room temperature for three hours. The annealing cycle was replicated, and the blocks were stored overnight at 4°C before sectioning.

Using a standard microtome sectioning technique, 4-μm sections were cut from TMA blocks and transferred into permanent positively charged glass slides (Surgipath, X-tra™ Adhesive, Peterborough, UK). To ensure the quality of the TMA construction, the first section of each block was stained with H&E and was scanned to be reviewed histologically.
