# Supplementary material for: Geometric characteristics of stromal collagen fibres in breast cancer using differential interference contrast microscopy
Source: J Microsc. 2024 Oct 3;297(2):135–52. doi: 10.1111/jmi.13361 (PMC11733853; doi:10.1111/jmi.13361)
Supplement: Supplementary file 3 — Supporting Information [file JMI-297-135-s003.docx]

**Association of stromal collagen characteristics with outcome in DCIS cohort**

No association of orientation angle (*p*<0.13), alignment (*p*<0.2), width (*p*<0.9), length (*p*<0.15), straightness (*p*<0.9) and density (0.9) with overall survival.

No association of orientation angle (*p*<0.78), alignment (*p*<0.44), width (*p*<0.47), length (*p*<0.87), straightness (*p*<0.34), and density (0.82) with local recurrence free interval (LRFI).

No association of orientation angle (*p*<0.21), alignment (*p*<0.29), width (*p*<0.25), length (*p*<0.25), straightness (*p*<0.32), and density (*p*<0.30) with distant metastasis free interval (DMFI).
