# Supplementary figures and images for: Geometric characteristics of stromal collagen fibres in breast cancer using differential interference contrast microscopy
Source: J Microsc. 2024 Oct 3;297(2):135–52. doi: 10.1111/jmi.13361 (PMC11733853; doi:10.1111/jmi.13361)

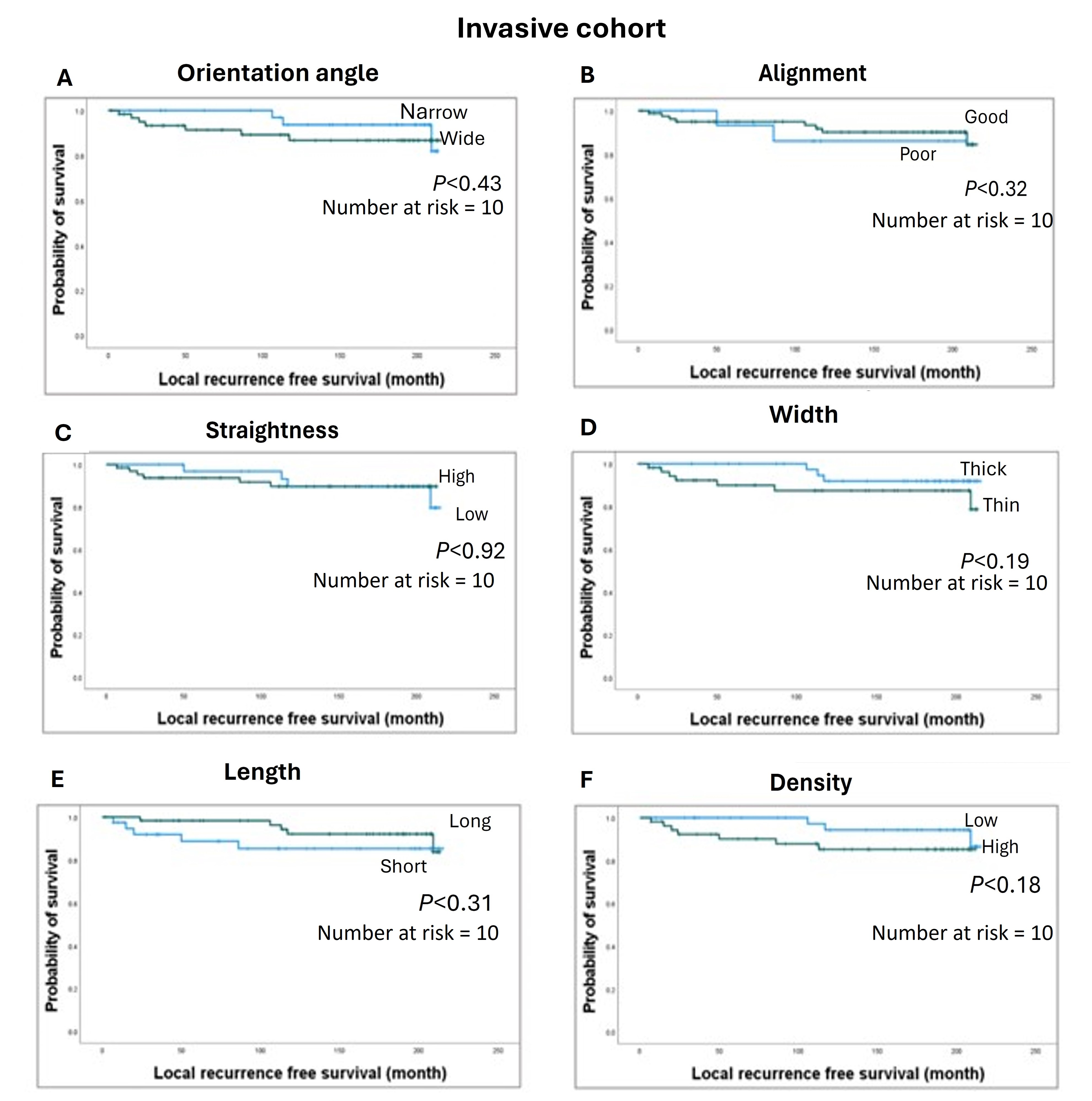

Supplement: Supplementary file 4 — Supporting Information [file JMI-297-135-s009.tiff]

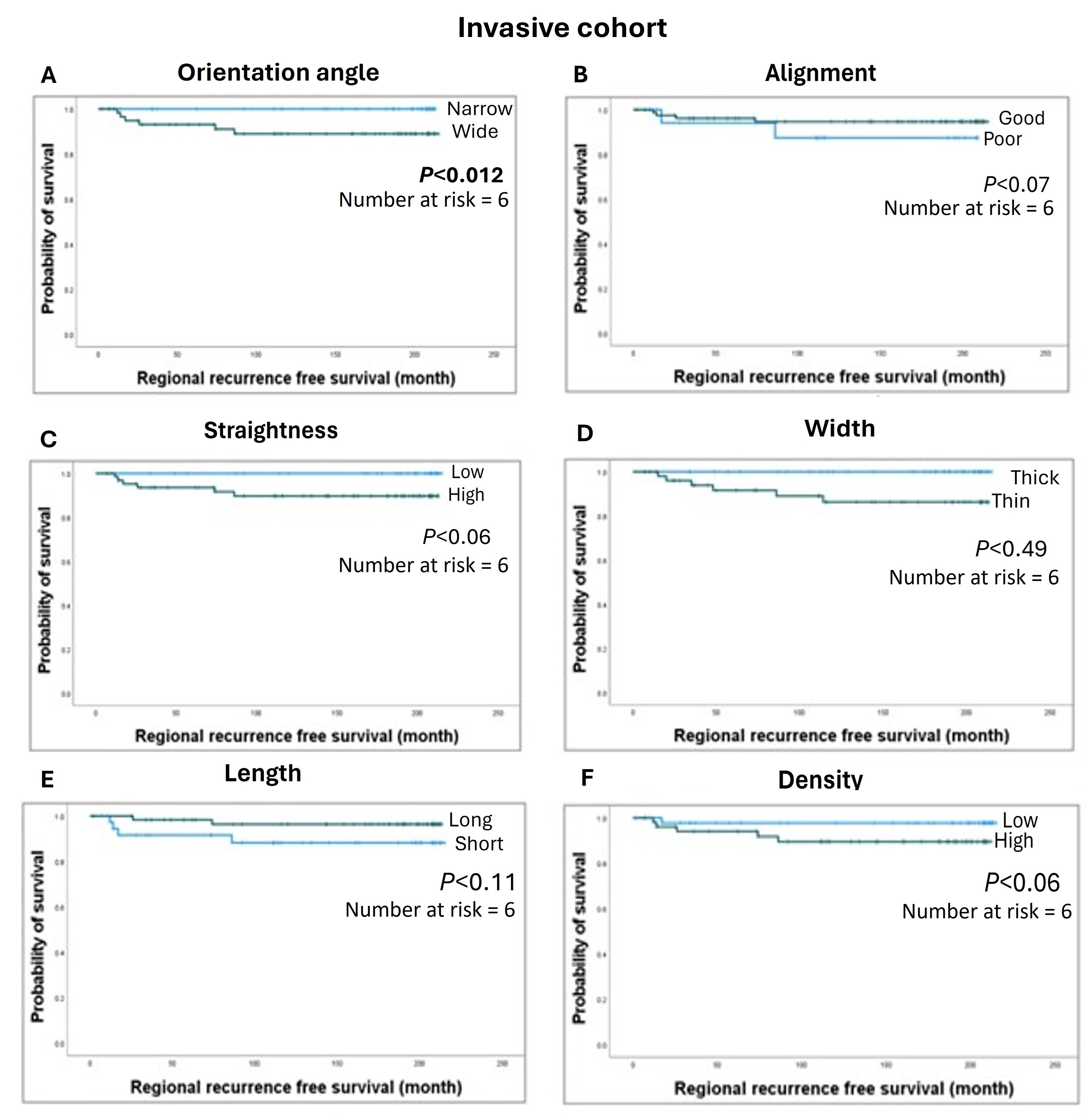

Supplement: Supplementary file 5 — Supporting Information [file JMI-297-135-s008.tiff]

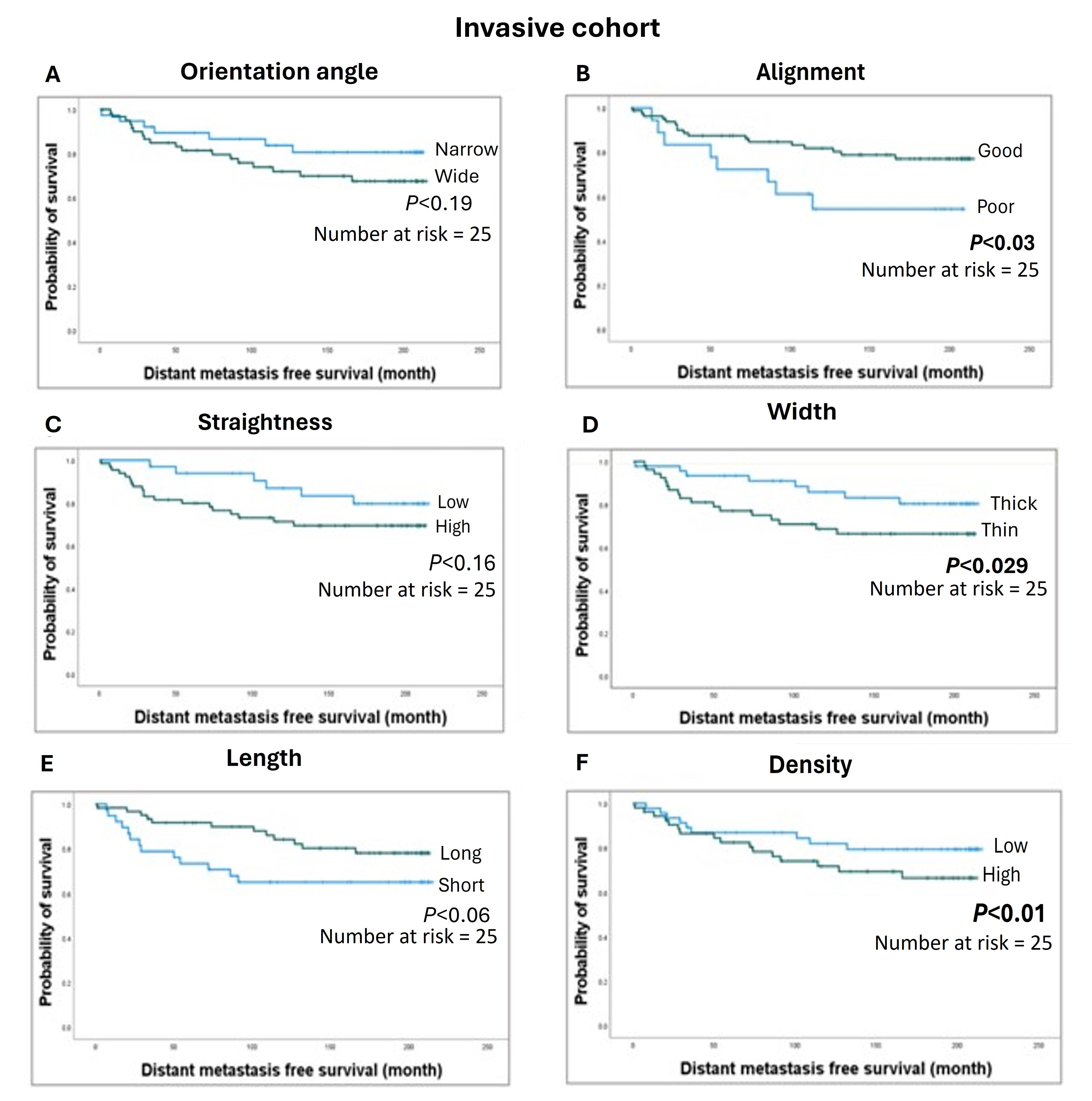

Supplement: Supplementary file 6 — Supporting Information [file JMI-297-135-s005.tiff]
