# Supplementary material for: Geometric characteristics of stromal collagen fibres in breast cancer using differential interference contrast microscopy
Source: J Microsc. 2024 Oct 3;297(2):135–52. doi: 10.1111/jmi.13361 (PMC11733853; doi:10.1111/jmi.13361)
Supplement: Supplementary file 7 — Supporting Information [file JMI-297-135-s007.docx]

**Supplementary Table 1.** Comparisons of the percentage of stromal collagen fibre characteristics between DCIS and invasive BC

|  | Orientation angle  (Degrees) | | Alignment  (Scale 0-1) | | Straightness  (Scale 0-1) | | Fibre width  (μm) | | Fibre length  (μm) | | Fibre density  (fibres/µm^2^) | |
| --- | --- | --- | --- | --- | --- | --- | --- | --- | --- | --- | --- | --- |
|  | **Narrow** | **Wide** | **Poor** | **Good** | **Low** | **High** | **Thin** | **Thick** | **Short** | **Long** | **Low** | **High** |
| DCIS cohort (N=100) | 56% | 44% | 51% | 49% | 46% | 54% | 50% | 50% | 52% | 48% | 46% | 54% |
| Invasive cohort  (N=100) | 38% | 62% | 82% | 18% | 34% | 66% | 54% | 46% | 39% | 61% | 40% | 60% |

Mean fibre length (defined as the end-to-end length of the fibre), fibre density (the concentration of collagen fibres in a given area), mean straightness (defined as the rate of curvature ranged from 0 more curve to 1 straighter), the mean orientation angle (angle between fibre and horizontal line resembling the duct border) and fibre alignment (the fibre direction compared to each other).
