# Supplementary material for: Geometric characteristics of stromal collagen fibres in breast cancer using differential interference contrast microscopy
Source: J Microsc. 2024 Oct 3;297(2):135–52. doi: 10.1111/jmi.13361 (PMC11733853; doi:10.1111/jmi.13361)
Supplement: Supplementary file 8 — Supporting Information [file JMI-297-135-s006.docx]

**Supplementary Table 2. Correlation of fibre directionality against clinicopathological data in the DCIS cohort.**

| **Parameter** | **Orientation angle**  **(Degrees)** | | | **Alignment**  **(Scale 0-1)** | | | **Straightness**  **(Scale 0-1)** | | |
| --- | --- | --- | --- | --- | --- | --- | --- | --- | --- |
|  | **Narrow** | **Wide** | ***P value*** | **Poor** | **Good** | ***P value*** | **Low** | **High** | ***P value*** |
| **Patient age**  <50 years  >50 years | 18(57%)  38(56%) | 14(43%)  30(44%) | *P*<0.97 | 18(56%)  33(48%) | 14(44%)  35(52%) | *P*<0.47 | 13(41%)  33(49%) | 19(59%)  35(51%) | *P*<0.45 |
| **Size**  < 2cm  >2cm | 20(69%)  36(51%) | 9(31%)  35(49%) | *P*<0.09 | 8(28%)  43(61%) | 21(72%)  28(39%) | ***P*<0.003*** | 21(72 %)  25(35%) | 8(28%)  46(65%) | ***P*<0.001*** |
| **Grade**  Low  Intermediate  High | 11(69%)  30(91%)  15(29%) | 5(31%)  3(9%)  36(71%) | ***P*<0.001*** | 0(0%)  2(6%)  49(96%) | 16(100%)  31(94%)  2(4%) | ***P*<0.001*** | 16(100%)  27(82%)  3(6%) | 0(0%)  6(18%)  48(94%) | ***P*<0.001*** |
| **Heterogeneity**  Absence  Presence | 52(55%)  4(80%) | 43(45%)  1(20%) | ***P*<0.026** | 47(49%)  4(80%) | 48(51%)  1(20%) | *P*<0.18 | 45(47%)  1(20%) | 50(53%)  4(80%) | *P*<0.23 |
| **Comedo necrosis type**  Absence  Presence | 28(70%)  28(47%) | 12(30%)  32(53%) | ***P*<0.021*** | 8(20%)  43(72%) | 32(80%)  17(28%) | ***P*<0.001*** | 31(77%)  15(25%) | 9(23%)  45(75%) | ***P*<0.001*** |
| **Type**  Not mixed  Mixed | 24(55%)  32(57%) | 20(45%)  24(43%) | *P*<0.79 | 23(52%)  28(50%) | 21(48%)  28(50%) | *P*<0.82 | 21(48%)  25(45%) | 23(52%)  31(55%) | *P*<0.75 |
| **Molecular subtypes**  Luminal A  Luminal B  Her2 enriched  TNBC | 47(75%)  2(29%)  1(20%)  1(11%) | 16(25%)  5(71%)  4(80%)  8(89%) | ***P*<0.001*** | 23(37%)  5(71%)  4(80%)  9(100%) | 40(63%)  2(29%)  1(20%)  0(0%) | ***P*<0.001*** | 40(63%)  2(29%)  1(20%)  0(0%) | 23(37%)  5(71%)  4(80%)  9(100%) | ***P*<0.001*** |
| **ER receptor status**  Negative  Positive | 2(13 %)  51(68%) | 13(87%)  24(32%) | ***P*<0.001*** | 14(93%)  30(40%) | 1(7%)  45(60%) | ***P*<0.001*** | 1(7%)  43(57%) | 14(93%)  32(43%) | ***P*<0.001*** |
| **PR receptor status**  Negative  Positive | 6(21%)  47(77%) | 23(79%)  14(23%) | ***P*<0.001*** | 25(86%)  19(31%) | 4(14%)  42(69%) | ***P*<0.001*** | 2(7%)  41(67%) | 27(93%)  20(33%) | ***P*<0.001*** |
| **HER receptor status**  Negative  Positive | 48(63.2%)  4(28.6%) | 28(36.8%)  10(71.4%) | ***P*<0.03*** | 36(47.4%)  10(71.4%) | 40(52.6%)  4(28.6%) | *P*<0.25 | 40(53%)  3(21%) | 36(47%)  11(79%) | ***P*<0.032*** |
| **Ki67 score**  Low  High | 49(65%)  2(14%) | 26(35%)  12(86%) | ***P*<0.001*** | 32(43%)  13(93%) | 43(57%)  1(7%) | ***P*<0.003*** | 41(55%)  1(7%) | 34(45%)  13(93%) | ***P*<0.001*** |

*** indicates *p*<0.05.**
