# Supplementary material for: Geometric characteristics of stromal collagen fibres in breast cancer using differential interference contrast microscopy
Source: J Microsc. 2024 Oct 3;297(2):135–52. doi: 10.1111/jmi.13361 (PMC11733853; doi:10.1111/jmi.13361)
Supplement: Supplementary file 9 — Supporting Information [file JMI-297-135-s010.docx]

**Supplementary Table 3. Correlation of fibre characteristics compared to the clinicopathological data in the DCIS cohort.**

| **Parameter** | **Fibre width (μm)** | | | **Fibre length (μm)** | | | **Fibre density (fibres/µm**^2^**)** | | |
| --- | --- | --- | --- | --- | --- | --- | --- | --- | --- |
|  | **Thin** | **Thick** | ***P value*** | **Short** | **Long** | ***P value*** | **Low** | **High** | ***P value*** |
| **Patient age**  <50 years  >50 years | 14(44%)  36(53%) | 18(56%)  32(47%) | *P*<0.93 | 15(47%)  37(54%) | 17(53%)  31(46%) | *P*<0.48 | 15(47%)  31(46%) | 17(53%)  37(54%) | *P*<0.90 |
| **Size**  < 2cm  >2cm | 22(76%)  28(39%) | 7(24%)  43(61%) | ***P*<0.001*** | 22(76%)  30(42%) | 7(24%)  41(58%) | ***P*<0.002*** | 19(66%)  27(38%) | 10(34%)  44(62%) | ***P*<0.012*** |
| **Grade**  Low  Intermediate  High | 16(100%)  26(79%)  8(16%) | 0(0%)  7(21%)  43(84%) | ***P*<0.001*** | 15(94%)  22(67%)  15(29%) | 1(6%)  11(33%)  36(71%) | ***P*<0.001*** | 16(100%)  27(82%)  3(6%) | 0(0%)  6(18%)  48(94%) | ***P*<0.001*** |
| **Heterogeneity**  Absence  Presence | 49(52%)  1(20%) | 46(48%)  4(80%) | *P*<0.16 | 47(49%)  5(100%) | 48(51%)  0(0%) | ***P*<0.028** | 45(47%)  1(20%) | 50(53%)  4(80%) | *P*<0.23 |
| **Comedo necrosis**  **type**  Absence  Presence | 31(78%)  19(32%) | 9(22%)  41(68%) | ***P*<0.001*** | 29(73%)  23(38%) | 11(27%)  37(62%) | ***P*<0.001*** | 31(78%)  15(25%) | 9(22%)  45(75%) | ***P*<0.001*** |
| **Type**  Not mixed  Mixed | 24(55%)  26(46%) | 20(45%)  30(54%) | *P*<0.42 | 27(61%)  25(45%) | 17(39%)  31(55%) | *P*<0. 09 | 20(46%)  26(46%) | 24(54%)  30(54%) | *P*<0.92 |
| **Molecular subtypes**  Luminal A  Luminal B  Her2 enriched TNBC | 42(67%)  2(29%)  1(20%)  1(11%) | 21(33%)  5(71%)  4(80%)  8(89%) | ***P*<0.001*** | 42(67%)  3(43%)  0(0%)  2(22%) | 21(33%)  4(57%)  5(100%)  7(78%) | ***P*<0.002*** | 38(60%)  2(28.6%)  1(20%)  0(0%) | 25(40%)  5(71%)  4(80%)  9(100%) | ***P*<0.001*** |
| **ER receptor status**  Negative  Positive | 2(13%)  45(60%) | 13(87%)  30(40%) | ***P*<0.002*** | 2(13%)  47(63%) | 13(87%)  28(37%) | ***P*<0.001*** | 1(7%)  42(56%) | 14(93%)  32(44%) | ***P*<0.001*** |
| **PR receptor status**  Negative  Positive | 4(14%)  41(67%) | 25(86%)  20(33%) | ***P*<0.001*** | 5(17%)  43(70%) | 24(83%)  18(30%) | ***P*<0.001*** | 3(10%)  41(67%) | 26(90%)  20(33%) | ***P*<0.001*** |
| **HER receptor status**  Negative  Positive | 43(57%)  3(21%) | 33(43%)  11(79%) | ***P*<0.043*** | 44(58%)  3(21%) | 32(42%)  11(79%) | ***P*<0.043*** | 38(50%)  4(29%) | 38(50%)  10(71%) | *P*<0.30 |
| **Ki67 score**  Low  High | 43(57%)  0(0%) | 32(43%)  14(100%) | ***P*<0.001*** | 44(59%)  3(21%) | 31(41%)  11(79%) | ***P*<0.034*** | 41(55%)  1(7%) | 34(45%)  13(93%) | ***P*<0.004*** |

*** indicates *p*<0.05.**
