# Supplementary material for: Geometric characteristics of stromal collagen fibres in breast cancer using differential interference contrast microscopy
Source: J Microsc. 2024 Oct 3;297(2):135–52. doi: 10.1111/jmi.13361 (PMC11733853; doi:10.1111/jmi.13361)
Supplement: Supplementary file 10 — Supporting Information [file JMI-297-135-s004.docx]

**Supplementary Table 4. Correlation of fibre directionality compared to the clinicopathological data in the invasive cohort.**

| **Parameter** | **Orientation angle**  **(Degrees)** | | | **Alignment**  **(Scale 0-1)** | | | **Straightness**  **(Scale 0-1)** | | |
| --- | --- | --- | --- | --- | --- | --- | --- | --- | --- |
|  | **Narrow** | **Wide** | ***P value*** | **Good** | **Poor** | ***P value*** | **Low** | **High** | ***P value*** |
| **Patient age**  <50 years  >50 years | 10 (36%)  28 (39%) | 18(64%)  44(61%) | *P*<0.76 | 20(71%)  62(86%) | 8(29%)  10(14%) | *P*<0.08 | 6(21)  28(39%) | 22(79%)  44(61%) | *P*<0.098 |
| **Tumour size**  < 2cm  >2cm | 23(36%)  15(43%) | 42(64%)  20(57%) | *P*<0.46 | 56(86%)  26(74%) | 9(14%)  9(26%) | *P*<0.14 | 30(46%)  4(11%) | 35(54%)  33(89%) | ***P*<0.001*** |
| **Tumour grade**  Grade 1  Grade 2  Grade 3 | 7(94%)  11(30%)  11(24%) | 1(6%)  26(70%)  34(76%) | ***P*<0.001*** | 18(100%)  36(97%)  28(62%) | 0(0%)  1(3%)  17(38%) | ***P*<0.001*** | 18(100%)  14(38%)  2(4%) | 0(0%)  23(62%)  43(96%) | ***P*<0.001*** |
| **Stage**  Stage I  Stage II  Stage III | 16(33%)  18(51%)  4(24%) | 32(66.7%)  17(48.6%)  13(76.5%) | *P*<0.09 | 40(83%)  29(83%)  13(77%) | 8(17%)  6(17%)  4(23%) | *P*<0.80 | 18(37%)  15(43%)  1(6%) | 30(63%)  20(57%)  16(94%) | ***P*<0.024*** |
| **NPI groups**  Good  Moderate  Poor | 11 (32%)  20 (46%)  7 (32%) | 23(68%)  24(54%)  15(68%) | *P*<0.39 | 33(97%)  35(79%)  14(64%) | 1(3%)  9(20%)  8(36%) | ***P*<0.005*** | 22(65%)  12(27%)  0(0%) | 12(35%)  32(73%)  22(100%) | ***P*<0.001*** |
| **LVI**  Negative  Definite | 28 (43%)  10 (29%) | 37(57%)  25(71%) | *P*<0.15 | 58(89%)  24(69%) | 7 (11%)  11(31%) | ***P*<0.01*** | 31(48%)  3(9%) | 34(52%)  32(91%) | ***P*<0.001*** |
| **Histological type**  NST  Lobular  Special | 24(36%)  3(50%)  11(39%) | 42(64%)  3(50%)  17(61%) | *P*<0.79 | 49(74%)  6(100%)  27(96%) | 17(26%)  0(0%)  1(4%) | ***P*<0.019*** | 15(23%)  3(50%)  16(57%) | 51(77%)  3(50%)  12(43%) | ***P*<0.004*** |
| **Molecular subtypes**  Luminal A  Luminal B  Her2 enriched TNBC | 14(41%)  17(45%)  2(29%)  1(10%) | 20(59%)  21(55%)  5(71%)  9(90%) | *P*<0.21 | 32(94%)  29(76%)  2(29%)  4(40%) | 2(6%)  9(24%)  5(71%)  6(60%) | *P*<0.055 | 20(59%)  10(26%)  0(0%)  1(10%) | 14(41%)  28(73%)  7(100%)  9(90%) | ***P*<0.001*** |
| **ER receptor status**  Negative  Positive | 3(17%)  35(43%) | 15(83%)  47(57%) | ***P*<0.039*** | 12(67%)  70(85%) | 6(33%)  12(15%) | *P*<0.06 | 1(6%)  33(40%) | 17(94%)  49(60%) | ***P*<0.005*** |
| **PR receptor status**  Negative  Positive | 14 (30%)  24 (45%) | 32(70%)  29(55%) | *P*<0.13 | 34(74%)  47(89%) | 12(26%)  6(11%) | *P*<0.057 | 10(22%)  24(45%) | 36(78%)  29(55%) | ***P*<0.014*** |
| **HER receptor status**  Negative  Positive | 31 (39%)  7 (33%) | 48(61%)  14(67%) | *P*<0.62 | 68(86%)  14(67%) | 11(14%)  7(33%) | ***P*<0.04*** | 31(39%)  3(14%) | 48(61%)  18(86%) | ***P*<0.032*** |
| **Ki67 score**  Low  High | 15 (39%)  17 (39%) | 23(61%)  26(61%) | *P*<0.99 | 35(92%)  33(77%) | 3(8%)  10(23%) | *P*<0.06 | 21(55%)  9(21%) | 17(45%)  34(79%) | ***P*<0.001*** |

*** indicates *p*<0.05.**
