# Supplementary material for: Geometric characteristics of stromal collagen fibres in breast cancer using differential interference contrast microscopy
Source: J Microsc. 2024 Oct 3;297(2):135–52. doi: 10.1111/jmi.13361 (PMC11733853; doi:10.1111/jmi.13361)
Supplement: Supplementary file 11 — Supporting Information [file JMI-297-135-s001.docx]

**Supplementary Table 5. Correlation of fibre characteristics compared to clinicopathological data in the invasive cohort.**

| **Parameter** | **Fibre width (μm)** | | | **Fibre length (μm)** | | | **Fibre density (fibres/µm**^2^**)** | | |
| --- | --- | --- | --- | --- | --- | --- | --- | --- | --- |
|  | **Thin** | **Thick** | ***P value*** | **Short** | **Long** | ***P value*** | **Low** | **High** | ***P value*** |
| **Patient age**  <50 years  >50 years | 11(39%)  43(59%) | 17(61%)  29(40%) | *P*<0.06 | 18(64%)  21(29%) | 10(36%)  51(71%) | ***P*<0.001*** | 11(39%)  29(40%) | 17(61%)  43(60%) | *P*<0.33 |
| **Tumour size**  < 2cm  >2cm | 42(64%)  12(34%) | 23(36%)  23(66%) | ***P*<0.004*** | 22(34%)  17(49%) | 43(66%)  18(51%) | *P*<0.15 | 38(58%)  2(6%) | 27(42%)  33(94%) | ***P*<0.004*** |
| **Tumour grade**  Grade 1  Grade 2  Grade 3 | 18(100%)  27(73%)  9(20%) | 0(0%)  10(22%)  36(80%) | ***P*<0.001*** | 0(0%)  6(16%)  33(73%) | 18(100%)  31(84%)  12(27%) | ***P*<0.001*** | 18(100%)  20(54%)  2(4%) | 0(0%)  17(46%)  43 (96%) | ***P*<0.001*** |
| **Stage**  Stage I  Stage II  Stage III | 31(65%)  21(60%)  2(12%) | 17(35%)  14(40%)  15(88%) | ***P*<0.001*** | 15(31%)  12(34%)  12(71%) | 33(69%)  23(66%)  5(29%) | ***P*<0. 01*** | 27(56%)  12(34%)  1(6%) | 21(44%)  23(66%)  16(94%) | ***P*<0.005*** |
| **NPI groups**  Good  Moderate  Poor | 31(91%)  20(46%)  3(14%) | 3 (9%)  24(54%)  19(86%) | ***P*<0.001*** | 3(9%)  19(43%)  17(77%) | 31(91%)  25(87%)  5(23%) | ***P*<0.001*** | 28(82%)  10(23%)  2(9%) | 6(18%)  34(77%)  20(91%) | ***P*<0.001*** |
| **LVI**  Negative  Definite | 43(66%)  11(31%) | 22(34%)  24(69%) | ***P*<0.001*** | 18(28%)  21(60%) | 47(72%)  14(40%) | ***P*<0.002*** | 35(54%)  5(14%) | 30(46%)  30(86%) | ***P*<0.004*** |
| **Histological type**  NST  Lobular  Mixed | 29(44%)  3(50%)  22(79%) | 37(56%)  3(50%)  6(21%) | ***P*<0.009*** | 32(49%)  3(50%)  4(14%) | 34(51%)  3(50%)  24(86%) | ***P*<0.007*** | 20(30%)  2(33%)  18(64%) | 46(70%)  4(67%)  10(36%) | ***P*<0.001*** |
| **Molecular subtypes**  Luminal A  Luminal B  Her2 enriched TNBC | 31(91%)  14(37%)  2(29%)  1(10%) | 3(9%)  24(63%)  5(71%)  9(90%) | ***P*<0.001*** | 2(6%)  20(52%)  5(71%)  8(80%) | 32(94%)  18(48%)  2(29%)  2(20%) | ***P*<0.001*** | 24(71%)  9(24%)  2(29%)  0(0%) | 10(30%)  29(76%)  5(71%)  9(100%) | ***P*<0.001*** |
| **ER receptor status**  Negative  Positive | 3(17%)  51(62%) | 15(84%)  31(38%) | ***P*<0.001*** | 14(78%)  25(31%) | 4(22%)  57(69%) | ***P*<0.001*** | 0(0%)  40(49%) | 18(100%)  42(51%) | ***P*<0.016*** |
| **PR receptor status**  Negative  Positive | 19(41%)  35(66%) | 27(86%)  18(34%) | ***P*<0.014*** | 24(52%)  14(26%) | 22(48%)  39(74%) | ***P*<0.009*** | 11(24%)  29(55%) | 35(76%)  24(45%) | *P*<0.18 |
| **HER receptor status**  Negative  Positive | 50(63%)  4(19%) | 29(37%)  17(81%) | ***P*<0.001*** | 25(32%)  14(67%) | 54(68%)  7(33%) | ***P*<0.003*** | 36(46%)  4(19%) | 43(44%)  17(81%) | ***P*<0.003*** |
| **Ki67 score**  Low  High | 32(84%)  15(35%) | 6(16%)  28(65%) | ***P*<0.001*** | 5(13%)  26(60%) | 33(87%)  17(40%) | ***P*<0.001*** | 27(71%)  15(35%) | 11(29%)  28(65%) | ***P*<0.001*** |

*** indicates *p*<0.05.**
